# Comparative efficacy and harms of adjuvant whole breast hypofractionation or external beam accelerated partial breast irradiation for treatment of breast cancer: a systematic review

**DOI:** 10.1186/s12885-026-16129-5

**Published:** 2026-06-25

**Authors:** Catherine Sowerby, Kassahun Lemu, Adrienne Landsteiner, Kristen Ullman, Maylen Anthony, Caleb Kalinowski, Elizabeth Ester, Philipp Dahm, Timothy J. Wilt, Wei Duan-Porter

**Affiliations:** 1 Center for Care Delivery & Outcomes Research, Minneapolis VA Health Care System, One Veterans Drive (152), Minneapolis, Minneapolis, MN 55417 USA; 2 Department of Orthopedic Surgery, University of Minnesota Medical School, Minneapolis, MN USA; 3 Department of Radiation Oncology, University of Minnesota Medical School, Minneapolis, MN USA; 4 Minneapolis VA Health Care System, Minneapolis, MN USA; 5 Department of Urology, University of Minnesota Medical School, Minneapolis, MN USA; 6 Department of Medicine, University of Minnesota School of Medicine, Minneapolis, MN United States; 7 Division of Health Policy & Management, University of Minnesota School of Public Health, Minneapolis, MN USA

**Keywords:** Hypofractionated radiation therapy, Skin toxicity, Pneumonitis, Lymphedema

## Abstract

**Background:**

Whole breast moderate hypofractionation is recommended for most breast cancer populations to improve survival and reduce recurrence. Whole breast ultra-hypofractionation and external beam accelerated partial breast irradiation (APBI) may reduce toxicity while maintaining benefits.

**Methods:**

MEDLINE and Embase searches through March 2024 identified randomized controlled trials (RCTs) evaluating adjuvant external beam hypofractionated radiation therapy for breast cancer. Eligibility and risk of bias (RoB) were assessed by two reviewers and required consensus. For prioritized outcomes (e.g., overall survival, acute toxicity), certainty of evidence was assessed using GRADE.

**Results:**

Thirty-nine RCTs compared whole breast moderate hypofractionation vs. conventional radiation (CFRT; *k* = 25); whole breast ultra-hypofractionation vs. moderate hypofractionation or CFRT (*k* = 8); or external beam APBI vs. whole breast radiation (*k* = 6). For all comparisons, there were low RoB, large trials (*N* > 2,000) with 5–10 year outcomes. Trials on ultra-hypofractionation and APBI focused on early stage cancer, some also including ductal carcinoma in situ (DCIS). High survival (> 90%) and low recurrence were found across all treatments. Moderate hypofractionation probably results in less overall acute toxicity, but makes little to no difference in specific adverse events. No trial on ultra-hypofractionation reported overall toxicity; evidence on acute skin toxicity is very uncertain. APBI probably results in less overall acute toxicity, but may result in more overall late toxicity; evidence was very uncertain or showed no difference for specific events.

**Conclusion:**

Radiation treatments had similar survival and recurrence. Moderate hypofractionation and APBI result in lower overall acute toxicity, but evidence is lacking for ultra-hypofractionation. For ultra-hypofractionation and APBI, future research is needed to better understand late toxicity outcomes, and whether effects may vary across settings and subgroups.

**Supplementary Information:**

The online version contains supplementary material available at https://doi.org/10.1186/s12885-026-16129-5.

## Introduction

Breast cancer is the most commonly diagnosed cancer and the second leading cause of cancer-related deaths among women globally [1, 2]. In the United States (US), the lifetime probability of developing breast cancer is one in eight women [3]. US projections for 2025 are 319,750 new cases and 42,680 deaths from breast cancer this year [3]. Radiation therapy is an essential component of effective treatment for breast cancer, and often follows either breast-conserving surgery or mastectomy [4]. Radiation therapy is important for improving long-term survival and reducing local recurrence, but there are significant concerns with resource costs, treatment access, and patient burden, particularly for conventional fractionation regimens [5, 6]. Whereas conventionally fractionated radiation therapy entails a total dose of 45.0–50.4 Gy delivered over 5–6 weeks, moderate hypofractionation has a condensed duration of 3–4 weeks (total dose 40.0–43.5 Gy), and ultra-hypofractionated regimens occur over even shorter courses of 1–2 weeks (total dose 26–30 Gy). Moderate hypofractionation has been noted to produce similar benefits for survival and local recurrence, and since 2018, has been recommended by the American Society for Radiation Oncology (ASTRO) for breast cancer treatment [6, 7]. To further reduce concomitant costs and/or access barriers, ultra-hypofractionated regimens and accelerated partial breast irradiation (APBI) have also been evaluated, particularly for early stage breast cancer. In addition to establishing the evidence for these radiation therapies in terms of long-term survival and local recurrence, we also need to better understand the evidence for relative risks for toxicity and harms.

In this paper, we present results addressing adjuvant external beam hypofractionated radiation treatment for breast cancer, which was part of a broader report conducted by the US Department of Veterans Affairs (VA) Evidence Synthesis Program (ESP) on the effects of hypofractionated radiation therapy for a variety of cancers [8]. We evaluated evidence from randomized controlled trials (RCTs) comparing various external beam radiation therapy regimens, including whole breast moderate hypofractionation and ultra-hypofractionation, and APBI. We summarize findings on survival, recurrence, and toxicity, organized by the following comparisons: whole breast moderate hypofractionation vs. conventional fractionation; whole breast ultra-hypofractionation vs. either moderate hypofractionation or conventional fractionation; and APBI vs. whole breast moderate hypofractionation or conventional fractionation. We then discuss evidence gaps and implications for clinical practice and policy.

## Methods

The prespecified protocol was registered with PROSPERO (CRD42021287645) and PRISMA Guidelines for systematic review reporting were followed (Supplemental Material 1).

### Scope and key questions

The US Veterans Health Administration (VHA) Radiation Oncology Quality Task Force requested a systematic review from VA ESP to inform development of clinical guidelines for use of external beam hypofractionation radiation therapy as curative treatment for a variety of cancer types. Radiation treatment with palliative intent (e.g., to manage pain and other symptoms) was not included. The VA ESP collaborated with stakeholders from the VHA Radiation Oncology Quality Task Force and an expert advisory panel to develop scope, key questions (KQs), and eligibility criteria. In this paper, we focus on those results (from the broader ESP report [8]) that address the following KQs specifically for breast cancer:

- KQ1: What are the comparative efficacy and harms of hypofractionation and conventional fractionation radiation therapy in the definitive treatment of adults with cancer?
- KQ2: How do the efficacy and harms of the above radiation regimens vary by cancer stage or other patient characteristics?

### Data sources and searches

For the VA ESP report, MEDLINE and Embase were searched from inception to January 5, 2022, for English-language articles using keywords and subject headings for radiation therapy, hypofractionation, and cancer (see **Supplemental Table 1** for exact search strategies). We updated the searches, specifically for studies addressing breast cancer, on March 12, 2024.

### Study selection

Search results were first processed and de-duplicated in Endnote, then uploaded into DistillerSR for further de-duplication, screening, and selection [9]. Using prespecified eligibility criteria (**Supplemental Table 2**), search results were screened by up to two reviewers for inclusion. Exclusion of articles required agreement by two reviewers, while articles included by at least one reviewer underwent full-text review. At full-text review, two individuals evaluated each article and reached consensus on final eligibility. Eligible articles reported RCTs evaluating external beam hypofractionation or conventional radiation therapy in at least one arm for the curative treatment of breast cancer and reported at least one of the eligible outcomes (i.e., overall and disease-free survival, local and regional recurrence, grade ≥ 2 toxicity [overall and specific], quality of life, and cost or resource use).

### Risk of bias assessment & data extraction

Two reviewers independently assessed risk of bias (RoB) for each trial using Cochrane Risk of Bias for Randomized Trials 2 (RoB 2) [10]. Reviewers reached consensus on overall ratings and domain scores, or involved a third reviewer as needed.

From all eligible studies, the following information was extracted by one reviewer and verified by a second reviewer: study characteristics (e.g., country, length of follow-up, funding source), participant characteristics (e.g., age, sex, cancer staging), intervention and comparator characteristics (e.g., radiation dose, fractions, and duration of treatment), and reported outcomes (e.g., overall and disease-specific survival, recurrence, toxicity, and quality of life). From studies rated low or some concerns for RoB, we further abstracted the detailed findings for all outcomes of interest.

### Synthesis

We analyzed findings separately for various comparisons: whole breast moderate hypofractionation vs. conventional fractionation; whole breast ultra-hypofractionation vs. either moderate hypofractionation or conventional fractionation; and APBI vs. whole breast moderate hypofractionation or conventional fractionation. We conducted meta-analyses when there were at least three trials (rated low RoB or some concerns) that were sufficiently similar with regard to interventions and comparators, and applicable outcomes. Meta-analyses used random effects models with the Hartung–Knapp–Sidik–Jonkman estimator to generate pooled effects, 95% confidence intervals (CI), and τ^2^. To visualize the impact of heterogeneity, we also present prediction intervals. All analyses were run in R v.4.2.1. When quantitative synthesis was not appropriate, we conducted qualitative syntheses.

With the assistance of the expert advisory panel and VA stakeholders, the following outcomes were prioritized for certainty of evidence (COE) assessments: overall survival, disease-free survival, local-regional recurrence, and acute and late toxicity (grade ≥ 2 according to Common Terminology Criteria for Adverse Events [CTCAE] and/or Radiation Therapy Oncology Group [RTOG] and European Organization for Research and Treatment of Cancer [EORTC] criteria). In addition to overall toxicity, skin toxicity and pneumonitis were also selected for COE assessments. We applied Grading of Recommendations, Assessment, Development and Evaluation (GRADE) criteria [11] for COE and used GRADEpro GDT [12] to help generate COE tables. With input from the advisory panel and stakeholders, the clinically important thresholds were defined for each outcome and applied to relevant GRADE domains (imprecision and inconsistency). The threshold was 5% absolute difference (between groups) for overall and disease-specific survival, and 10% absolute difference for local-regional recurrence over any length of follow-up. The threshold for grade ≥ 2 toxicity (overall or specific events) was 10% absolute difference.

## Results

### Overview

**Fig. 1:**
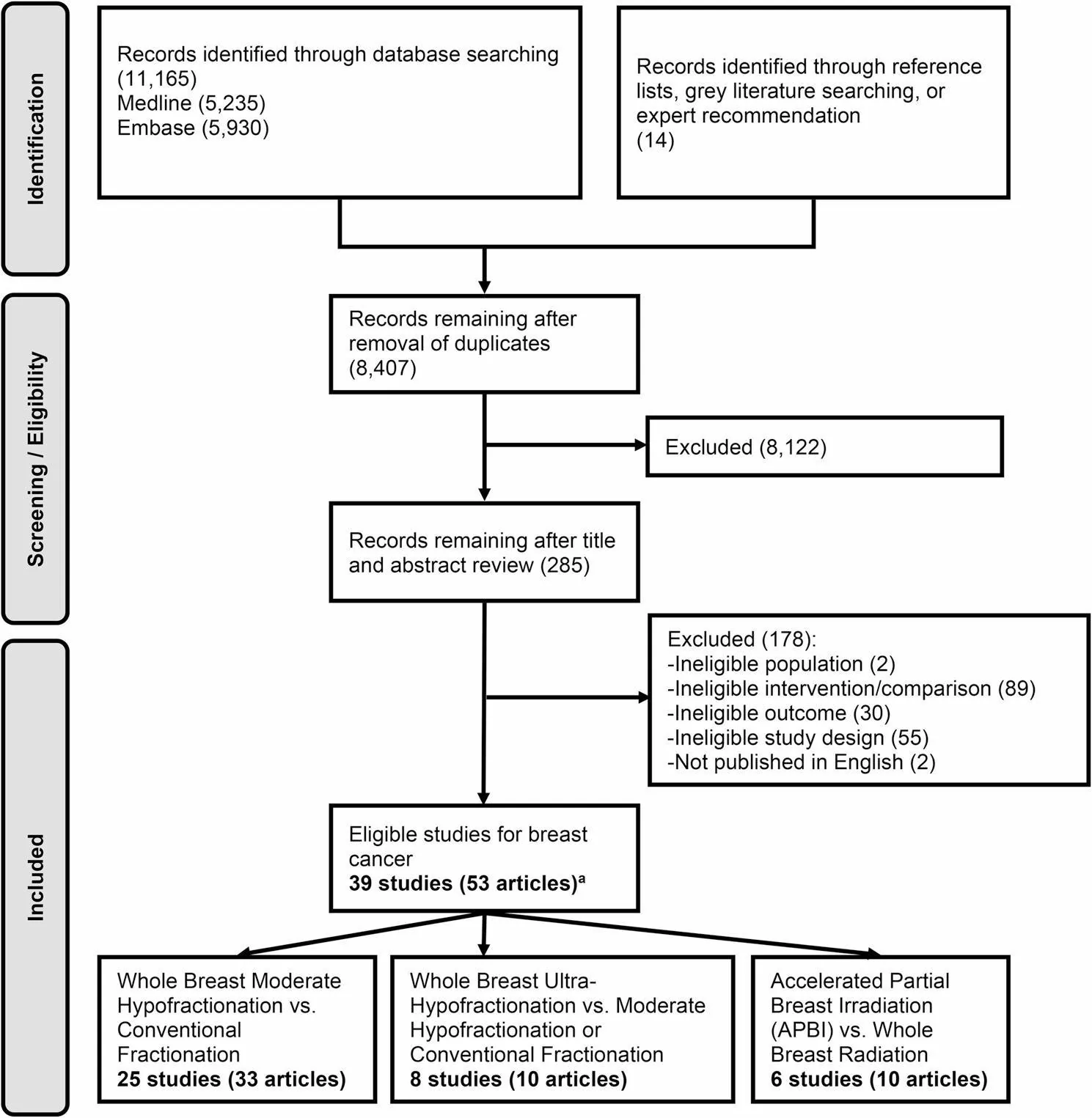
Search & selection of eligible studies. ^a^39 studies were described by 53 eligible articles on breast cancer (out of 107 total eligible articles addressing all cancers)

We identified 39 eligible trials (reported in 53 publications) addressing effects of adjuvant hypofractionated radiation therapy for treatment of breast cancer (Fig. 1). The majority of trials compared moderate hypofractionation with conventional whole breast radiation therapy (*k* = 25 studies), and fewer evaluated ultra-hypofractionated whole breast radiation (*k* = 8) or external beam APBI (*k* = 6). Table 1 summarizes the characteristics of all eligible trials, categorized by radiation treatment comparisons and RoB ratings. Half of the trials were rated high RoB (*k* = 19), with a similar proportion of high RoB studies across different radiation treatment comparisons (Table 1). Detailed RoB ratings for each study are provided in **Supplemental Table 3**. Most trials enrolled more than 100 participants (*k* = 30) and a third had more than 500 people (*k* = 13). Participants were middle-aged or older women, and half of studies focused on early stage invasive breast cancer and/or ductal carcinoma in situ (DCIS) (*k* = 20), including all trials evaluating APBI. Trials were conducted mostly in Asian or European countries, and less commonly in Africa or South America. Only one trial was conducted in the United States. A third of trials had follow-up greater than 5 years (*k* = 15). Trials most often evaluated survival and toxicity, while fewer reported on quality of life and only one trial [13] examined costs (Table 1).

**Table 1:** Summary characteristics of eligible breast cancer trials

Study Characteristics	Whole Breast Moderate Hypofractionation vs. Conventional Fractionation		Whole Breast Ultra-Hypofractionation^a^		Accelerated Partial Breast Irradiation (APBI)^b^	
	Low/Mod ROB (k = 12)	High ROB (k = 13)	Low/Mod ROB (k = 5)	High ROB (k = 3)	Low/Mod ROB (k = 3)	High ROB (k = 3)
*Sample Size*						
0-100	--	7	1	--	1	--
101–500	4	6	2	3	--	2
501–1000	2	--	1	--	1	1
> 1000	6	--	1	--	1	--
*Age*,* mean or median*						
40–55	--	8	--	1	--	1
56–70	6	2	4	1	1	1
> 70	--	--	--	--	--	--
NR^c^	6	3	1	1	2	1
*Tumor Stage*						
I-II	6^d^	5	3	1	3^e^	3
I-III	4	4	2	1	--	--
II-III	--	4	--	1	--	--
III	1	--	--	--	--	--
DCIS only	1^f^	--	--	--	--	--
*Study Location*						
Africa	--	1	--	--	--	--
Asia	2	10	1	1	--	1
Europe	6	--	4	1	2	2
North America	2	1	--	1	--	--
South America	--	1	--	--	--	--
Multiple Countries	2	--	--	--	1	--
*Length of Follow-up*						
< 1 year	1	2	1	--	--	--
1 to < 5 years	1	9	2	1	1	3
≥ 5 years	10	1	2	1	2	--
Unclear	--	1	--	1	--	--
*Survival Outcomes*						
Overall survival	7	3	3	2	3	2
Disease-free survival	6	3	1	2	1	2
Locoregional recurrence	6	4	2	3	1	--
Local recurrence	6	2	2	--	2	1
*Harms Outcomes*						
Overall toxicity (grade ≥ 2)	--	3	--	--	2	--
Acute skin toxicity	5	9	4	2	3	2
Acute pneumonitis	2	--	--	--	2	--
Late skin toxicity	3	5	1	1	2	2
Late pneumonitis	1	1	--	--	1	--
Late lymphedema	3	2	--	2	--	--
* Quality of Life*	3	2	2	1	1	1
* Cost or Resource Use*	--	--	--	--	--	1

*Abbreviatons:APBI *Accelerated partial breast irradiation, *DCIS *Ductal carcinoma in situ, *RCT *Randomized controlled trial, *ROB *Risk of bias

^a^6 trials compared ultra-hypofractionation with moderate hypofractionation [14–19], and 2 trials with conventional fractionation [20, 21]

^b^4 trials compared APBI with whole breast moderate hypofractionation [13, 22–24], and 2 trials with whole breast conventional radiation [25–27]

^c^Trials did not report age or only provided descriptives for age categories

^d^2 trials also included participants with DCIS [28–30]

^e^1 trial also included participants with DCIS [24]

^f^One trial included participants with DCIS, meeting criteria for “increased risk of recurrence” [31]

For comparing efficacy and harms of these different radiation treatments, we focused on findings from the 20 trials that were low or some concerns for RoB. These trials tended to be larger (> 500 participants) and had longer follow-up (≥ 5 years), compared with high RoB studies (Table 1). Table 2 provides detailed study and participant characteristics for these 20 trials. Below we present detailed results and synthesis of findings from these studies, grouped by different comparisons of radiation treatment regimens, beginning with moderate hypofractionation vs. conventional radiation therapy.

**Table 2:** Detailed characteristics of eligible breast cancer trials (rated low or some concerns for risk of bias)

Trial Name or Trial Registration Number Risk of Bias Country Funding Follow-up	Participant Eligibility Criteria Other Treatments	Intervention Arm(s) Characteristics			Comparator Arm Characteristics	Primary Outcome Other Outcomes Reported^a^
		Dose/fraction; Total Dose; Treatment Duration *N* randomized Baseline characteristics			Dose/fraction; Total Dose; Treatment Duration *N* randomized Baseline characteristics	
Moderate Hypofractionation vs. Conventional Fractionation (Whole Breast Radiation)						
**BIG3-07/TROG 07.01** NCT00470236 (King, 2020 (31)) SOME CONCERNS 11 countries (118 sites) National Health and Medical Research Council, Susan G. Komen for the Cure, Breast Cancer Now, OncoSuisse Federation Against Cancer, Dutch Cancer Society 2 years	**Inclusion**: women ≥ 18 years old with completely excised DCIS and increased risk of local recurrence (age < 50 years, or in those ≥ 50 years old, symptomatic presentation, palpable tumor, tumor ≥ 15 mm, multifocal disease, intermediate or high nuclear grade, central necrosis, comedo histology, and/or radial surgical margin < 10 mm **Exclusion**: NR **Other treatments**: • Radiation boost • Endocrine therapy	2.66 Gy/fraction; 42.5 Gy; 3.5 weeks *N* = 532 ≥ 50 years old: 445 (84%) Race: NR Tumor information NR			2.0 Gy/fraction; 50 Gy; 5 weeks *N* = 615 ≥ 50 years old: 495 (80%) Race: NR Tumor information NR	**Primary**: Local recurrence (NR)^b^ **Quality of Life**
**DBCG HYPO** NCT00909818 (Offersen, 2020 (30)) LOW Denmark, Germany, and Norway (8 sites) Danish Cancer Society, Centre for Interventional Research in Radiation Oncology, and Danish Comprehensive Cancer Center Radiotherapy Group 9 years	**Inclusion**: Women > 40 years old, had breast-conserving surgery without immediate reconstruction, early stage (pT1-2N0-1M0) or DCIS, requiring radiotherapy to residual breast only **Exclusion**: Need for regional lymph node radiation, previous breast cancer or bilateral, past radiation of thorax or breast, breast implants, comorbidity which may increase sensitivity to radiation (e.g., dermatomyositis, or symptomatic heart/lung disease), or any difficulty completing protocol **Other treatments**: • Radiation boost • Chemotherapy • Endocrine therapy • Trastuzumab	2.67 Gy/fraction; 40 Gy; 3 weeks *N* = 917 Age, median (range): 59 (41–82) Race: NR DCIS: 123 (13%) T1a: 64 (8%) T1b: 191 (24%) T1c: 403 (51%) T2: 136 (17%) N0: 683 (86%) N1: 76 (10%) Isolated tumor cells: 35 (4%)			2.0 Gy/fraction; 50 Gy; 5 weeks *N* = 937 Age, median (range): 59 (42–83) Race: NR DCIS: 123 (13%) T1a: 48 (6%) T1b: 196 (24%) T1c: 414 (51%) T2: 156 (19%) N0: 661 (81%) N1: 107 (13%) Isolated tumor cells: 46 (6%)	**Primary**: Cosmetic (breast induration at 3 years) **Survival & Recurrence**: • Overall • Locoregional recurrence
DRKS00017763 (Schmeel, 2020 (32)) SOME CONCERNS Germany (multicenter) Funding NR 6 weeks	**Inclusion**: Women > 18 years old, had breast-conserving surgery **Exclusion**: metastatic disease, chemotherapy, need for regional nodal irradiation, previous radiation to ipsilateral breast, breast-reconstruction or any previous surgery in radiation area, active smoking, active skin condition, use of topical or oral corticosteroids, tattoos in the irradiation area Other treatments: NR	2.67 Gy/fraction; 40.05 Gy; duration NR *N* = 71 Age, mean (SD): 59.9 (± 10.7) Race: Caucasian 70 (99%) T1: 48 (68%) T2: 16 (23%)			2.0 Gy/fraction; 50 Gy; duration NR *N* = 72 Age, mean (SD): 59.0 (± 11.7) Race: Caucasian 70 (97%) T1: 43 (60%) T2: 16 (23%)	**Primary**: Dermatitis, grade ≥ 2
NCT00156052 (Whelan, 2010; (33) Whelan, 2002; (34) Arsenault, 2020; (35) Goldberg, 2023 (36)) LOW Canada (8 sites) Canadian Breast Cancer Research alliance, Canadian Cancer Society 12 years median follow-up	**Inclusion**: women with invasive breast cancer, had lumpectomy and negative axillary lymph nodes **Exclusion**: cancer involving margins of excision, tumor > 5 cm, breast width > 25 cm Other treatments • Chemotherapy • Endocrine therapy	2.66 Gy/fraction; 42.5 Gy; 22 days *N* = 622 ≥ 60 years old: 277 (45%) Race: NR *Tumor grade*: I: 215 (35%) II: 244 (39%) III: 117 (19%) Unknown: 46 (7%)			2.0 Gy/fraction; 50 Gy; 35 days *N* = 612 ≥ 60 years old: 309 (51%) Race: NR *Tumor grade*: I: 209 (34%) II: 236 (39%) III: 116 (19%) Unknown: 51 (8%)	**Primary**: Local recurrence **Survival & Recurrence**: • Overall • Disease-free • Local recurrence **Harms**: • Skin toxicity, acute & late (some concerns) **Quality of life** (high)
NCT00793962 (Wang, 2019 (37)) LOW China (1 site) National Key Projects of Research and Development of China, Chinese Academy of Medical Science Innovation Fund for Medical Sciences, and Beijing Marathon of Hope, Cancer Foundation of China 5 years (median follow-up 59.5 months)	**Inclusion**: women 18–75 years old, had mastectomy and axillary dissection with negative margins and ≥ 4 positive axillary lymph nodes or primary T3/4 disease; Karnofsky score ≥ 60% **Exclusion**: bilateral breast cancer, positive supraclavicular or internal mammary node, distant metastasis, had breast reconstruction or previous radiation, had past or current other cancer, or other serious comorbidity (drug use, mental illness, collagen vascular disease, etc.) Other treatments: • Chemotherapy • Endocrine therapy • Trastuzumab	2.9 Gy/fraction; 43.5 Gy; 3 weeks *N* = 406 ≥ 50 years old: 194 (48%) Race: NR *Overall stage*: III: 377 (94%) *Tumor grade*: III: 121 (30%)			2.0 Gy/fraction; 50 Gy; 5 weeks *N* = 414 ≥ 50 years old: 202 (49%) Race: NR *Overall stage*: III: 384 (94%) *Tumor grade*: III: 111 (27%)	**Primary**: Locoregional recurrence **Survival** • Overall • Disease-free **Harms**: • Skin toxicity, acute & late • Pneumonitis, acute • Lymphoedema, late
NCT01266642 (Shaitelman, 2015; (28) Shaitelman, 2018; (29) Weng, 2021 (38)) SOME CONCERNS US (1 site) American Society of Clinical Oncology, Breast Cancer Research Foundation, Cancer Prevention and Research Institute of Texas, University of Texas MD Anderson Cancer Center, gift from Ann and Clarence Cazalot, and NCI 5 years	**Inclusion**: women ≥ 40 years, DCIS or stage I-II breast cancer (Tis-T2, N0-N1a, M0), breast-conserving surgery with negative margins (defined as “no tumor on ink”) and no need for third field to cover regional lymph nodes **Exclusion**: ongoing treatment for another cancer, past breast cancer, bilateral breast cancer, prior overlapping irradiation, or lack of fluency in English or Spanish. Other treatments: • Boost radiation • Chemotherapy	2.66 Gy/fraction; 42.56 Gy; duration NR *N* = 138 ≥ 50 years old: 119 (86%) Race: White: 99 (72%) Hispanic: 20 (15%) Black: 17 (12%) Asian: 2 (1%) DCIS: 24 (17%) pN0: 95 (69%) pN1mic: 6 (4%) pN1a: 7 (5%) *Tumor grade*: I: 34 (25%) II: 73 (53%) III: 30 (22%)			2.0 Gy/fraction; 50 Gy; duration NR *N* = 149 ≥ 50 years old: 136 (92%) Race: White: 116 (78%) Hispanic: 16 (11%) Black: 15 (10%) Asian: 2 (1%) DCIS: 39 (26%) pN0: 101 (68%) pN1mic: 14 (9%) pN1a: 1 (1%) *Tumor grade*: I: 40 (27%) II: 70 (47%) III: 39 (26%)	**Primary**: cosmetic (3 years) **Survival** (high): • Overall • Local recurrence **Harms**: • Overall toxicity, acute & late • Skin toxicity, acute & late • Pneumonitis, late • Lymphedema, late **Quality of life**
NCT01413269 (Wang, 2020 (39)) LOW China (4 sites) Chinese Academy of Science 5 years (median follow-up 73.5 months)	**Inclusion**: Women 18–70 years old with invasive breast cancer, T1/2 disease, had undergone lumpectomy and axillary dissection (or sentinel node biopsy if sentinel nodes were negative) with negative margins (microscopically tumor-free ≥ 1 mm) **Exclusion**: supraclavicular/ internal mammary node or distant metastasis, received neoadjuvant chemotherapy, bilateral breast cancer, or had undergone previous irradiation or malignancies Other treatments: • Chemotherapy	2.9 Gy/fraction; 43.5 Gy; 3 weeks (+ boost 8.7 Gy in 3 fractions over 3 days) *N* = 365 ≥ 45 years old: 216 (??) Race: NR *Overall stage*: I: 247 (68%) II: 106 (29%) III: 12 (3%) *Tumor grade*: I-II: 228 (63%) III: 101 (28%) Unknown: 36 (??)			2.0 Gy/fraction; 50 Gy; 5 weeks (+ boost 10 Gy in 5 fractions over 1 week) *N* = 364 ≥ 45 years old: 223 (??) Race: NR *Overall stage*: I: 248 (68%) II: 104 (29%) III: 12 (3%) *Tumor grade*: I-II: 248 (72%) III: 82 (23%) Unknown: 34 (??)	**Primary**: Local recurrence **Survival & Recurrence**: • Overall • Disease-free Locoregional recurrence **Harms**: • Skin toxicity, acute • Pneumonitis, acute • Lymphedema, late • Lung fibrosis, late
**START A** ISRCTN59368779 (START Trialists, 2008; (40) Haviland, 2013 (41)) LOW UK (17 sites) Cancer Research UK, UK Medical Research Council, Department of Health Median follow-up 9.3 years	**Inclusion**: women ≥ 18 years, invasive breast cancer (pT1-3a pN0-1M0) requiring radiotherapy after BCS or mastectomy with clear tumor margins ≥ 1 mm and no immediate reconstruction **Exclusion**: NR Other treatments: • Chemotherapy • Endocrine therapy	**Arm A**: 3.2 Gy/fraction; 41.6 Gy; 5 weeks *N* = 750 Age, mean (SD): 57.0 (± 10.7) Race: NR *Tumor size (cm)*: < 1: 26 (4%) 1-: 347 (46%) 2-: 203 (27%) 3-: 169 (23%) Not known: 5 (1%) : N0: 536 (72%) N1: 197 (26%) Not known: 17 (2%) *Tumor Grade*: I: 150 (20%) II: 379 (51%) III: 207 (28%)	**Arm B**: 3.0 Gy/fraction; 39 Gy; 5 weeks *N* = 737 Age, mean (SD): 57.1 (± 10.5) Race: NR *Tumor size (cm)*: < 1: 24 (3%) 1-: 355 (48%) 2-: 198 (27%) 3-: 157 (21%) Not known: 3 (0.3%) N0: 497 (67%) N1: 224 (30%) Not known: 16 (2%) *Tumor Grade*: 1: 149 (20%) 2: 368 (50%) 3: 210 (29%)		2.0 Gy/fraction; 50 Gy; 5 weeks *N* = 749 Age, mean (SD): 57.6 (± 10.5) Race: NR *Tumor size (cm)*: < 1: 24 (3%) 1-: 362 (48%) 2-: 202 (27%) 3-: 156 (21%) Not known: 5 (1%) N0: 514 (69%) N1: 222 (30%) Not known: 13 (2%) *Tumor Grade*: I: 157 (21%) II: 369 (49%) III: 212 (28%)	**Primary**: Locoregional recurrence **Survival & Recurrence**: • Overall • Disease-free • Local recurrence • Distant metastasis
**START B** ISRCTN59368779 (START Trialists, 2008; (42) Haviland, 2013 (41)) LOW UK (23 sites) Cancer Research UK, UK Medical Research Council, Department of Health Median follow-up 9.9 years	**Inclusion**: women ≥ 18 years, invasive breast cancer (pT1-3a pN0-1M0) requiring radiotherapy after BCS or mastectomy with clear tumor margins ≥ 1 mm and no immediate reconstruction **Exclusion**: NR Other treatments: • Chemotherapy • Endocrine therapy	2.67 Gy/fraction; 40.05 Gy; 3 weeks *N* = 1110 Age, mean (SD): 57.8 (± 9.5) Race: NR *Tumor size (cm)*: < 1: 167 (15%) 1-: 542 (49%) 2-: 288 (26%) 3-: 107 (10%) Not known: 6 (0.5%) N0: 804 (72%) N1: 266 (24%) Not known: 40 (4%) *Tumor Grade*: I: 311 (28%) II: 532 (48%) III: 248 (22%)			2.0 Gy/fraction; 50 Gy; 5 weeks *N* = 1105 Age, mean (SD): 57.0 (± 10.4) Race: NR *Tumor size (cm)*: < 1: 151 (14%) 1-: 552 (50%) 2-: 287 (26%) 3-: 113 (10%) Not known: 2 (0.2%) N0: 831 (75%) N1: 238 (22%) Not known: 36 (3%) *Tumor Grade*: I: 306 (28%) II: 518 (47%) III: 261 (24%)	**Primary**: Locoregional recurrence **Survival & Recurrence**: • Overall • Local recurrence • Distant metastasis • Disease-free
**START Pilot** Trial # NR (Owen, 2006 (43)) SOME CONCERNS UK (2 sites) Marks and Spencer PLC, Cancer Research UK 10 years	**Inclusion**: < 75 years old, operable invasive breast cancer (T1-3, N0/1, M0), had breast-preserving surgery and complete macroscopic resection **Exclusion**: NR Other treatments: • Radiation boost (2 Gy/fraction x 7) • Chemotherapy • Endocrine therapy	**Arm A**: 3.3 Gy/fraction; 42.9 Gy; 5 weeks *N* = 466 Demographics and tumor information NR (by arm)	**Arm B**: 3 Gy/fraction; 39 Gy; 5 weeks *N* = 474 Demographics and tumor information NR (by arm)		2 Gy/fraction; 50 Gy; 5 weeks *N* = 470 Demographics and tumor information NR (by arm)	**Primary**: Cosmetic (late change in breast appearance) **Survival & Recurrence**: • Local recurrence
**Trial Name/# NR** (Spooner, 2012 (44)) SOME CONCERNS UK (3 sites) Cancer Research UK 15 years (median follow-up 16.9 years)	**Inclusion**: women with stage I/II breast cancer, had complete surgical resection, tumor < 5 cm, no clinically palpable axillary nodes, no systemic disease **Exclusion**: past cancer, or history of radiation or chemotherapy Other treatments: • Chemotherapy • Tamoxifen (all)	2.66 Gy/fraction; 40 Gy; 3 weeks *N* = 181 Age, median (IQR): 59 (48–66) for whole group, NR by arm Race: NR Tumor information NR (by arm)			2.0 Gy/fraction; 50 Gy; 5 weeks *N* = 177 Age, median (IQR): 59 (48–66) for whole group, NR by arm Race: NR Tumor information NR (by arm)	**Primary**: locoregional recurrence (at 5 years) **Survival & Recurrence**: • Overall • Disease-free
**TomoBreast** NCT00459628 (Van Parijs, 2012; (45) Versmessen, 2012; (46) Van Parijs, 2023 (47)) SOME CONCERNS Belgium (1 site) Foundation against Cancer 12 years median follow-up	**Inclusion**: Women ≥ 18 years old, stage I-II (T1-3N0M0 or T1-2N1M0), had BCS or mastectomy with clear margins and axillary node dissection or sentinel node biopsy, had pre-operative imaging (CT, MRI, and/or PET) **Exclusion**: past breast or thoracic radiation, psychiatric or addictive disorder Other treatments • Boost radiation • Chemotherapy • Endocrine therapy	2.8 Gy/fraction; 42 Gy; 3 weeks *N* = 59 Age, mean (SD): 55 (± 11) Race: NR T1: 39 (66%) T2: 20 (34%) N0: 38 (64%) N1, LNR 0.01–0.20: 18 (86%) N1, LNR 0.21–0.65: 3 (14%) N1, LNR > 0.65: 0 *Tumor Grade*: I: 11 (30%) II: 18 (49%) III: 8 (22%) Unknown: 0			2.0 Gy/fraction; 50 Gy; 5 weeks *N* = 62 Age, mean (SD): 58 (± 11) Race: NR T1: 38 (61%) T2: 24 (39%) N0: 46 (74%) N1, LNR 0.01–0.20: 11 (69%) N1, LNR 0.21–0.65: 5 (31%) N1, LNR > 0.65: 0 *Tumor Grade*: I: 11 (34%) II: 8 (25%) III: 10 (31%) Unknown: 3	**Primary**: Cardiopulmonary function changes (at 3 years) **Survival & Recurrence**: • Overall • Disease-free **Harms**: • Skin toxicity, acute **Quality of life**
***Ultra-Hypofractionation vs. Moderate Hypofractionation or Conventional Radiation***						
**FAST** NCT00107497 (Brunt, 2020; (48) FAST Trialists, 2011 (20)) LOW UK (18 sites) National Health Service, Cancer Research UK/Institute of Cancer 10 years	**Inclusion**: Women ≥ 50 years old, had BCS, tumor < 3.0 cm, complete microscopic resection of tumor, and negative axillary node status **Exclusion**: Mastectomy, lymphatic radiotherapy, tumor bed boost dose and neoadjuvant or adjuvant chemotherapy Other treatments: • Endocrine therapy	**Arm A**: 6.0 Gy/fraction; 30 Gy; 5 weeks *N* = 308 Age, mean (SD): 62.9 (± 7.5) Race: NR *Tumor size (cm)*: < 1: 84 (30%) 1–2: 165 (54%) ≥ 2: 59 (19%) *Tumor Grade*: I: 113 (37%) II: 159 (52%) III: 35 (11%) Unknown: 1 (0.3%)	**Arm B**: 5.7 Gy/fraction; 28.5 Gy; 5 weeks *N* = 305 Age, mean (SD): 62.7 (± 6.8) Race: NR *Tumor size (cm)*: < 1: 87 (29%) 1–2: 160 (53%) ≥ 2: 58 (19%) *Tumor Grade*: I: 102 (33%) II: 168 (55%) III: 34 (11%) Unknown: 1 (0.3%)		2.0 Gy/fraction; 50 Gy; 5 weeks *N* = 302 Age, mean (SD): 63.1 (± 7.2) Race: NR *Tumor size (cm)*: < 1: 90 (30%) 1–2: 166 (55%) ≥ 2: 46 (15%) *Tumor Grade*: I: 94 (31%) II: 176 (58%) III: 29 (10%) Unknown: 3 (1%)	**Primary**: Cosmetic (change in breast appearance at 2 years) **Survival & Recurrence**: • Overall • Local recurrence • Regional metastasis • Distant metastasis • Breast cancer-specific deaths **Harms**: • Skin toxicity, acute
**FAST-Forward** ISRCTN19906132 (Brunt, 2020; (14) Brunt, 2016 (49)) LOW UK (97 sites) National Institute for Health Research, Cancer Research UK 5 years (median follow-up 71.5 months)	**Inclusion**: women ≥ 18 years old with stage pT1-3 pN0-1 M0 breast cancer, had BCS or mastectomy, axillary staging and/or dissection, and complete microscopic excision of primary tumor **Exclusion**: contralateral breast cancer, or past cancer (except if non-breast malignancy was treated with curative intent and ≥ 5 years disease free), breast reconstruction using implants, concurrent chemotherapy, or radiation to any regional lymph node areas (except lower axilla included in tangential fields to breast/chest wall) Other treatments: • Chemotherapy • Endocrine therapy • Trastuzumab	**Arm A**: 5.4 Gy/fraction; 27 Gy; 1 week *N* = 1367 Age, median (IQR): 61 (53, 67) Race: NR T1mi: 5 (0.4%) T1a: 68 (5.0%) T1b: 270 (19.8%) T1c: 601 (44.0%) T2: 389 (28.5%) T3: 30 (2.2%) Unknown: 4 (0.3%) N0: 1124 (82.2%) N1: 243 (17.8%) Unknown: 0		**Arm B**: 5.2 Gy/fraction; 26 Gy; 1 week *N* = 1368 Age, median (IQR): 61 (52, 66) Race: NR T1mi: 6 (0.4%) T1a: 51 (3.7%) T1b: 256 (18.7%) T1c: 602 (44.0%) T2: 424 (31.0%) T3: 25 (1.8%) Unknown: 4 (0.3%) N0: 1110 (81.1%) N1: 256 (18.7%) Unknown: 2 (0.1%)	2.67 Gy/fraction; 40 Gy; 3 weeks *N* = 1361 Age, median (IQR): 60 (53, 66) Race: NR T1mi: 4 (0.3%) T1a: 69 (5.1%) T1b: 258 (19.0%) T1c: 612 (45.0%) T2: 394 (28.9%) T3: 31 (1.5%) Unknown: 3 (0.2%) N0: 1103 (81.0%) N1: 257 (18.9%) Unknown: 1 (0.1%)	**Primary**: Local recurrence **Survival & Recurrence**: • Overall • Locoregional recurrence • Distant metastases **Harms** (some concerns): • Skin toxicity, acute
**HYPORT** NCT03788213 (Chakraborty, 2022 (17)) LOW India (3) Tata Medical Center, NAG foundation 7 months	**Inclusion**: women ≥ 18 years old, mastectomy (Cambridge score ≥ 3) or BCS with clear margins and adequate axillary clearance or sentinel lymph node biopsy **Exclusion**: DCIS, non-epithelial breast cancer, metaplastic carcinomas, residual IMC or supraclavicular fossa node, prior radiation therapy, concurrent chemotherapy **Other treatments**: • Endocrine therapy • Anti-HER2 therapy	5.2 Gy/fraction; 26 Gy; 1 week (+ boost 6 Gy in 5 fractions in some participants) *N* = 135 Age: NR Race: NR *Tumor Grade*: I: 3 (2.2%) II: 33 (24.4%) III: 99 (75%) II + III (bilateral): 1 (0.7%)			2.67 Gy/fraction; 40 Gy; 3 weeks (+ boost 8 Gy in 15 fractions in some participants) *N* = 136 Age: NR Race: NR *Tumor grade*: I: 6 (4.4%) II: 30 (22.05%) III: 100 (73.5%) II + III (bilateral): 0	**Primary**: Locoregional recurrence (NR)^b^ **Survival & Recurrence**: • Overall (NR)^b^ • Disease-free survival (NR)^b^ **Harms**: • Skin toxicity, acute **Quality of life** (NR)^b^
**Trial Name NR** (Ivanov, 2022 (18)) SOME CONCERNS Serbia (1) None 18 months	**Inclusion**: women > 40 years old, stage T1-3/N0-1/M0, treated with BCS and complete macroscopic resection of invasive carcinoma **Exclusion**: planned sequential boost, or indication for nodal treatment Other treatments: NR	5.2 Gy/fraction; 26 Gy; 1 week *N* = 27 Age, mean (SD): 62.8 (± 8.6) Race: NR *Overall stage*: I: 11 (40.7%) II: 16 (59.3%)			2.67 Gy/fraction; 40 Gy; 3 weeks *N* = 33 Age, mean (SD): 63.6 (± 9.8) Race: NR *Overall stage*: I: 13 (39.4%) II: 20 (60.6%)	**Primary**: NR **Harms**: • Skin toxicity, acute & late
**YO-HAI5** NCT03677427 (Van Hulle, 2021 (15)) SOME CONCERNS Belgium (1 site) University Hospital, Ghent 2–4 weeks	**Inclusion**: women ≥ 18 years old, treated with BCS and adjuvant whole breast radiation (± boost) **Exclusion**: lymph node metastases or distant metastases; bilateral breast irradiation or history of radiation to the same region; life expectancy < 2 years; planned reconstructive surgery; conditions making toxicity evaluation difficult (e.g., skin disorders); inability to follow protocol **Other treatments**: • Chemotherapy • Endocrine therapy • Trastuzumab	5.7 Gy/fraction; 28.5 Gy; 10–12 days *N* = 106 Age, median (range): 59 (37–83) Race: NR T1N0M0: 86 (81%) T1N1(mi)M0: 4 (4%) T2N0M0: 11 (10%) TisN0M0: 5 (5%)			2.67 Gy/fraction; 40.05 Gy; 10–12 days *N* = 94 Age, median (range): 62 (26–84) Race: NR T1N0M0: 77 (82%) T1N1(mi)M0: 2 (2%) T2N0M0: 7 (7%) TisN0M0: 8 (9%)	**Primary**: Cosmetic (breast retraction at 2 years) **Harms**: • Skin toxicity, acute **Quality of life**
***Accelerated Partial Breast Irradiation (APBI) vs. Whole Breast Hypofractionation or Conventional Regimen***						
**APBI-IMRT Florence** NCT02104895 (Meattini, 2015; (50) Livi, 2015; (25) Meattini, 2020 (51)) SOME CONCERNS Italy (1 site) Funding: none Median follow-up 10.7 years	**Inclusion**: women > 40 years old with early stage cancer (tumor ≤ 2.5 cm) suitable for BCS **Exclusion**: past cancer solid Tumor); history cardiovascular disease (e.g., heart failure, angina); FEV_1_ <1 L/m; extensive intraductal carcinoma; multiple foci cancer; final surgical margins < 5 mm; or absence of surgical clips in tumor bed. Other treatments: • Chemotherapy • Endocrine therapy	APBI-IMRT: 6.0 Gy/fraction; 30 Gy; 2 weeks *N* = 260 ≥ 60 years: 168 (61%) pTis: 23 (9%) pT1a: 28 (11%) pT1b: 98 (38%) pT1c: (97 (37%) pT2: 14 (5%) N0: 241 (89%) N1: 19 (7%) Unknown: 9 (4%) *Tumor Grade*: I: 124 (48%) II: 110 (38%) III: 26 (10%)			Whole Breast: 2.0 Gy/fraction; 50 Gy; duration NR (+ boost 2 Gy/fraction x 5 fractions) *N* = 260 ≥ 60 years: 139 (53%) pTis: 32 (12%) pT1a: 18 (7%) pT1b: 88 (34%) pT1c: 107 (41%) pT2:15 (6%) N0: 229 (82%) N1: 31 (13%) Unknown: 14 (5%) *Tumor Grade*: I: 103 (40%) II: 124 (48%) III: 33 (13%)	**Primary**: Local recurrence **Survival & Recurrence**: • Overall • Locoregional recurrence • Distant metastasis • Breast cancer-specific **Harms** • Overall toxicity, acute & late • Skin toxicity, acute & late
NCT06007118 (Burkon, 2024 (23)) SOME CONCERNS Czech Republic (1 site) Ministry of Health of the Czech Republic Median follow-up 37 months	**Inclusion**: Women ≥ 50 years old with DCIS (≤ 25 mm) or invasive ductal carcinoma (≤ 20 mm) who had BCS and negative margins, negative sentinel node biopsy or axillary dissection (≥ 6 negative nodes) **Exclusion**: absence of surgical clips in tumor bed, multifocal or multicentric involvement, prior chest or breast surgery, prior RT, adjuvant chemotherapy Other treatments: • Endocrine therapy	APBI-VMAT: 6.0 Gy/fraction; 30 Gy; 5–7 days *N* = 42 Age, median (range): 65 (52, 77) Race: NR DCIS: 2 (4.8%) *Tumor Size (mm)*: 0–9: 24 (57%) 10–14: 13 (31%) 15–20: 5 (12%) *Tumor Grade*: I: 25 (60%) II: 17 (40%)			Whole Breast: 2.67 Gy/fraction; 40.05 Gy; 4 weeks (+ boost 10 Gy in 5 fractions) *N* = 42 Age, median (range): 64 (51, 81) Race: NR DCIS: 2 (4.8%) *Tumor Size (mm)*: 0–9: 18 (43%) 10–14: 17 (40%) 15–20: 7 (17%) *Tumor Grade*: I: 24 (57%) II: 18 (43%)	**Primary**: Toxicity **Survival & Recurrence**: • Overall (NR)^b^ • Disease-free (NR)^b^ **Harms**: • Skin toxicity, acute & late • Pneumonitis, acute & late • Lymphedema, acute **Quality of life** (NR)^b^
**RAPID** NCT00282035 (Whelan, 2019; (24) Olivotto, 2013 (52)) SOME CONCERNS 3 countries (33 sites) Canadian Institutes for Health Research, Canadian Breast Cancer Research Alliance Median follow-up 8.6 years	**Inclusion**: Women ≥ 40 years old with DCIS or invasive ductal carcinoma who had BCS with microscopically clear margins, and negative axillary nodes (by sentinel node biopsy or axillary dissection for invasive cancer, or clinical exam for DCIS) **Exclusion**: tumor > 3 cm, lobular carcinoma, > 1 primary breast tumor Other treatments: • Chemotherapy • Endocrine therapy	APBI: 3.85 Gy/fraction; 38.5 Gy; 5–8 days (87% 3DCRT, 10% IMRT) *N* = 1070 ≥ 50 years old: 939 (88%) Race: NR DCIS: 191 (18%) Invasive cancer: 879 (82%) *Tumor size (cm)*: < 1.5: 613 (70%) ≥ 1.5: 266 (30%) pN0: 874 (99%) pNi+,pNMi: 5 (< 1%) *Tumor Grade* I: 387 (44%) II: 353 (40%) III: 133 (15%) Unknown: 6 (1%)			Whole Breast: 82% received: 2.65 Gy/fraction; 42.5 Gy; 4–5 weeks 18% received: 2 Gy/fraction; 50 Gy; 4–5 weeks (+ boost in 21%, 10 Gy in 4–5 fractions) *N* = 1065 ≥ 50 years old: 939 (88%) Race: NR DCIS: 190 (18%) Invasive cancer: 875 (82%) *Tumor size (cm)*: < 1.5: 587 (67%) ≥ 1.5: 288 (33%) pN0: 865 (99%) pNi+,pNMi: 10 (1%) *Tumor Grade* I: 362 (41%) II: 361 (41%) III: 143 (16%) Unknown: 9 (1%)	**Primary**: Local recurrence **Survival & Recurrence**: • Overall • Disease-free **Harms**: • Overall toxicity, acute & late • Skin toxicity, acute • Pneumonitis, acute

*Abbreviatons:3DCRT *Three-dimensional conformal radiation therapy, *APBI *Accelerated partial breast irradiation, *BCS *Breast-conserving surgery, *CT *Computed tomography, *DCIS *Ductal carcinoma in situ, *ECOG *Eastern Cooperative Oncology Group, *HFRT *Hypofractionated radiation therapy, *IMRT *Intensity-modulated radiation therapy, *MRI *Magnetic resonance imaging, *NR  *Not reported, *PET *Positron emission tomography, *SD *Standard deviation, *TNM  *TNM Classification of Malignant Tumors, *UK *United Kingdom, *US *United States, *VMAT *Volumetric modulated arc therapy

^a^Risk of bias noted separately if different by outcome

^b^Article(s) did not report findings for outcomes but stated these were being evaluated

### Whole breast moderate hypofractionation vs. conventional fractionation radiation therapy

Twelve trials (rated low or some concerns RoB) compared moderate hypofractionation, which consisted of 40.0–43.5 Gy delivered over 3–5 weeks (13–16 treatments of 2.65–2.9 Gy per fraction), with conventional regimens (total dose 50.0 Gy over 5 weeks, 2.0 Gy per fraction) (Table 2). In all groups, surgery preceded radiation treatment, and medication therapies (e.g., endocrine therapy, trastuzumab) were usually included at some point. Half of the trials focused on early stage breast cancer (*k* = 6) [28–32, 44–46, 53], with two of these also including participants with DCIS [28–30, 53]. One trial focused solely on those with DCIS with at least one high-risk factor [31]. Remaining trials included more advanced stage cancer in addition to early disease (stages I–III, *k* = 5) [33–35, 39–43] and one trial only included those with stage III cancer [37]. The largest trials were the Standardisation of Breast Radiotherapy (START) trials A [40, 41] and B [41, 42] (*N* = 2,327 and 2,236, respectively), both conducted in the United Kingdom (UK) from 1999 to 2012 (median follow-up 9.3 and 9.9 years). Only 2 trials had follow-up less than 5 years [31, 32]. About half of trials had local or local-regional recurrence as the primary outcome (*k* = 7) [31, 33–35, 37, 39–42, 44], while the remaining trials were designed to examine differences in cosmetic outcomes (*k* = 3) [28–30, 43, 53] or treatment toxicity (*k* = 2) [32, 45, 46]. Detailed findings for all eligible outcomes are provided in **Supplemental Table 4**.

#### Survival & recurrence outcomes

**Fig. 2:**
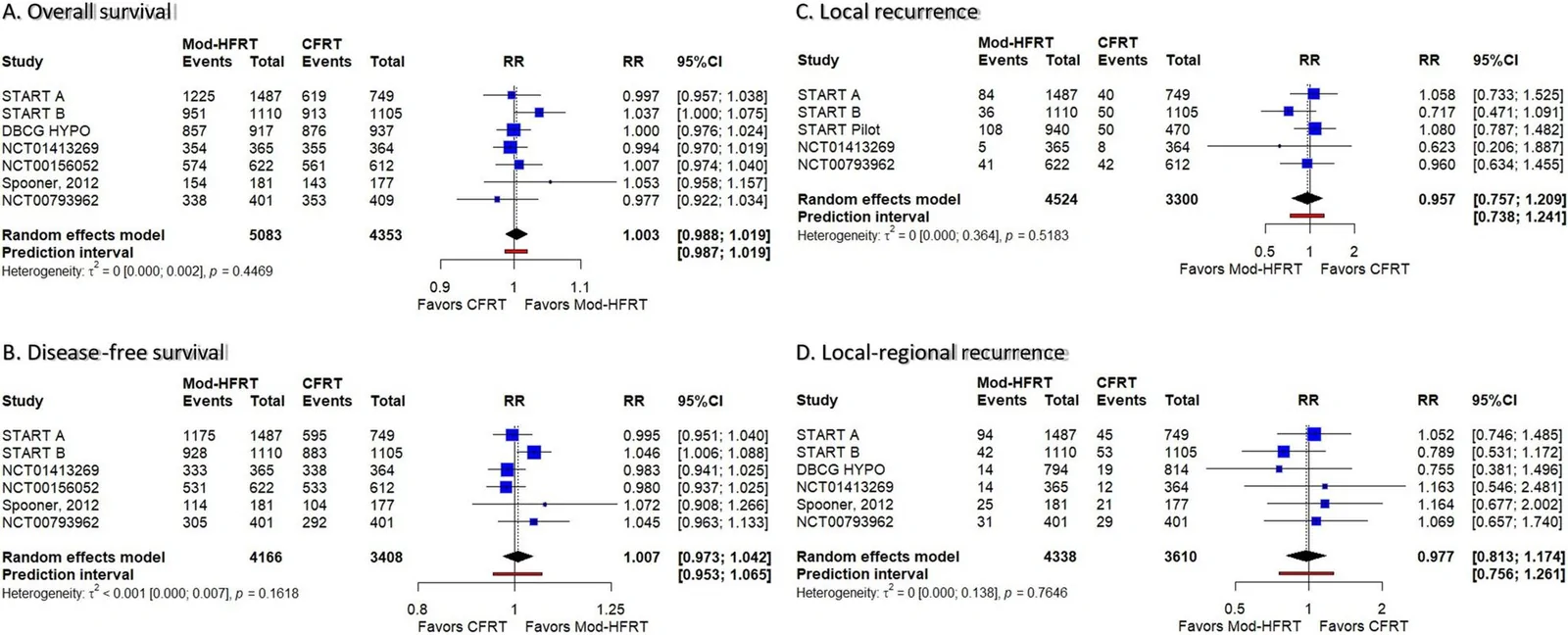
Survival & Recurrence Meta-analyses: Whole Breast Moderate Hypofractionation (Mod-HFRT) vs. Conventional Fractionation Radiation Therapy (CFRT). Abbreviations. CFRT=conventional fractionation radiation therapy; CI=confidence interval; Mod-HFRT=whole breast moderate hypofractionation; RR=risk ratio

Moderate hypofractionation results in little to no difference in overall survival, disease-free survival, or local-regional recurrence, compared with conventional regimens (high COE for all outcomes; Table 3). Seven trials evaluated overall survival [30, 33–35, 37, 39–42, 44] and most also reported disease-free survival, local recurrence, and local-regional recurrence over 6–10 years (Fig. 2). As noted above, the largest of these trials were START A and B, which included women with stage I-III breast cancer. Pooled estimates of relative risk (RR) ranged from 0.98 to 1.01 for survival and recurrence outcomes, with 95% CIs crossing 1 for all outcomes (Fig. 2). In both moderate hypofractionation and conventional radiation groups across all studies, overall survival was high (e.g., > 80% at 10 years) and recurrence rates were low (e.g., < 10% at 10 years) (**Supplemental Table 4**).

**Table 3:** Certainty of Evidence for Survival & Toxicity Outcomes: Whole Breast Moderate Hypofractionation (Mod-HFRT) vs. Conventional Fractionation Radiation Therapy (CFRT)

Outcome and MCID^a^	Follow-up No. of Participants (Studies)	Relative Effect (95% CI)	Anticipated Absolute Effects (95% CI)			Certainty	What Happens
			Mod-HFRT	CFRT	Difference		
**Overall survival** Absolute effect estimates based on control event rates at 6 and 10 years from START B trial (42) **Threshold: 5% difference**	5–10 years *N* = 9436 (7 RCTs) (30, 33, 34, 37, 39–42, 44)	**RR = 1.00** (0.99, 1.02)	**87.8%** (86.5, 89.2)	87.5%	*6 years*: **0.3% more** (1.1 fewer to 1.7 more)	⨁⨁⨁⨁ High	Moderate hypofractionation results in little to no difference in overall survival.
			**82.9%** (81.6, 84.2)	82.6%	*10 years*: **0.2% more** (1 fewer to 1.6 more)		
**Disease-free survival** Absolute effect estimates based on control event rates at 6 and 10 years from START B trial (42) **Threshold: 5% difference**	5–10 years *N* = 7574 (6 RCTs) (34, 37, 39–42, 44)	**RR = 1.01** (0.97, 1.04)	**85.8%** (82.9, 88.7)	85.2%	*6 years*: **0.6% more** (2.3 fewer to 3.6 more)	⨁⨁⨁⨁ High	Moderate hypofractionation results in little to no difference in disease-free survival.
			**80.5%** (77.8, 83.3)	79.9%	*10 years*: **0.6% more** (2.2 fewer to 3.4 more)		
**Local-regional recurrence** Absolute effect estimates based on control event rates at 6 and 10 years from START B trial (42) **Threshold: 10% difference**	5–10 years *N* = 7948 (6 RCTs) (30, 37, 39–42, 44)	**RR = 0.98** (0.81, 1.17)	**3.2%** (2.6, 3.8)	3.3%	*6 years*: **0.1% fewer** (0.6 fewer to 0.6 more)	⨁⨁⨁⨁ High	Moderate hypofractionation results in little to no difference in local-regional recurrence.
			**4.7%** (3.9, 5.6)	4.8%	*10 years*: **0.1% fewer** (0.9 fewer to 0.8 more)		
**Any acute toxicity** **(grade ≥ 2)** Risk ratio and absolute effects at ≤ 3 months from trial NCT01266642 (28, 29, 53) **Threshold: 10% difference**	3 months *N* = 287 (1 RCT) (28)	**RR = 0.61** (0.50, 0.74)	**47.1%** (35.0, 59.2)	78%	**30.8% fewer** (39.2 fewer to 20.6 fewer)	⨁⨁⨁◯ Moderate^a^	Moderate hypofractionation probably results in less overall acute toxicity.
**Any late toxicity** **(grade ≥ 2)** Risk ratio and absolute effects at 6 months from trial NCT01266642 (28, 29, 53) **Threshold: 10% difference**	6 months *N* = 271 (1 RCT) (28)	**RR = 0.96** (0.67, 1.36)	**31.0%** (16.7, 45.3)	32%	**1.4% fewer** (12.5 fewer to 9.7 more)	⨁⨁◯◯ Low^a, b^	Moderate hypofractionation may result in little to no difference in overall late toxicity.
**Acute skin toxicity** **(grade ≥ 2)** Absolute effect estimates based on control event rate at 3 months from trial NCT01413269 (39) **Threshold: 10% difference**	3 months *N* = 1370 (5 RCTs) (28, 32, 35, 39, 45)	**RR = 0.56** (0.31, 0.999)	**4.1%** (2.3, 7.4)	7.4%	**3.3% fewer** (5.1 fewer to 0 fewer)	⨁⨁◯◯ Low^a, c^	Moderate hypofractionation may result in little to no difference in acute skin toxicity.
**Late skin toxicity** **(grade ≥ 2)** Risk ratio and absolute effects at 5 and 10 years from trial NCT00156052 (33–35) **Threshold: 10% difference**	5–10 years *N* = 2054 (2 RCTs) (33, 37)	**RR = 0.94** (0.46, 1.96)	**3.1%** (1.5, 6.5)	3.3%	*5 years*: **0.2% fewer** (1.8 fewer to 3.2 more)	⨁⨁◯◯ Low^a, d^	Moderate hypofractionation may result in little to no difference in late skin toxicity.
		**RR = 1.16** (0.63, 2.13)	**8.9%** (4.8, 16.5)	7.7%	*10 years*: **1.2% fewer** (2.9 fewer to 8.8 more)		
**Acute pneumonitis** **(grade ≥ 2)** Risk ratio and absolute effects at 3 months from trial NCT01413269 (39) **Threshold: 10% difference**	6 months *N* = 1549 (2 RCTs) (37, 39)	**RR = 0.63** (0.25, 1.61)	**1.9%** (0.8, 4.9)	3.0%	**1.1% fewer** (2.3 fewer to 1.9 more)	⨁⨁⨁⨁ High	Moderate hypofractionation results in little to no difference in acute pneumonitis.

*Abbreviatons: CFRT *Conventional fractionation radiation therapy, *CI *Confidence interval, *HFRT *Hypofractionation radiation therapy, *NR  *Not reported, *RCT *Randomized controlled trial, *RR *Risk ratio

^a^MCIDs are absolute between-group differences in rates

**GRADE Working Group Certainly of Evidence Ratings**:

High certainty: we are very confident that the true effect lies close to that of the estimate of the effect

Moderate certainty: we are moderately confident in the effect estimate: the true effect is likely to be close to the estimate of the effect, but there is a possibility that it is substantially different

Low certainty: our confidence in the effect estimate is limited: the true effect may be substantially different from the estimate of the effect

Very low certainty: we have very little confidence in the effect estimate: the true effect is likely to be substantially different from the estimate of effect

**Explanations**:

a. Downgraded one level for study limitations (studies with some concerns for risk of bias)

b. Downgraded one level for imprecision (CI crossing clinical threshold in one direction)

c. Downgraded one level for inconsistency (substantial heterogeneity across studies)

d. Downgraded one level for indirectness (1 of 2 studies included grade 1 adverse event)

Five of the trials evaluating survival and recurrence also performed subgroup analyses to assess moderation by a variety of patient and disease characteristics [30, 33, 36, 39–43]. The largest analyses used combined data from the 3 START trials (A, B, and pilot; *N* = 5,861) [41], and showed no differences in treatment effects comparing moderate hypofractionation with conventional radiation, for different participant age groups, types of primary surgery, axillary node status, tumor grade, adjuvant chemotherapy use, or use of tumor bed boost. Similarly, the other trials also found that treatment effects on local or local-regional recurrence did not differ across a variety of factors, including patient age, cancer stage, and tumor size [30, 33, 36, 39], although one of these reported that moderate hypofractionation may be less effective for grade III tumors (hazard ratio [HR] 3.08, 95% CI [1.22, 7.76] for local recurrence, test for interaction *p* = 0.01) [33]. For participants with grade III tumors, the local recurrence rate at 10 years was 15.6% for the moderate hypofractionation group and 4.7% for the conventional radiation group [33].

#### Toxicity & harms

Moderate hypofractionation probably results in less overall acute toxicity, but has little to no difference in late toxicity at 6 months, compared with conventional fractionation (moderate and low COE, respectively; Table 3). Only 1 trial (*N* = 287) reported on overall toxicity, finding that grade ≥ 2 acute toxicity (within 42 days) was lower in the moderate hypofractionation group (47% [65/138]) vs. those who had conventional radiation therapy (78% [116/149]; *p* < 0.001) [28]. Late toxicity was assessed at 6 months and there were no differences in grade ≥ 2 events (hypofractionation group 31% [40/129] vs. conventional group 32% [46/142]; *p* = 0.81). This trial included women with DCIS or invasive cancer stage I-II, and evaluated toxicity using the CTCAE [28].

**Fig. 3:**
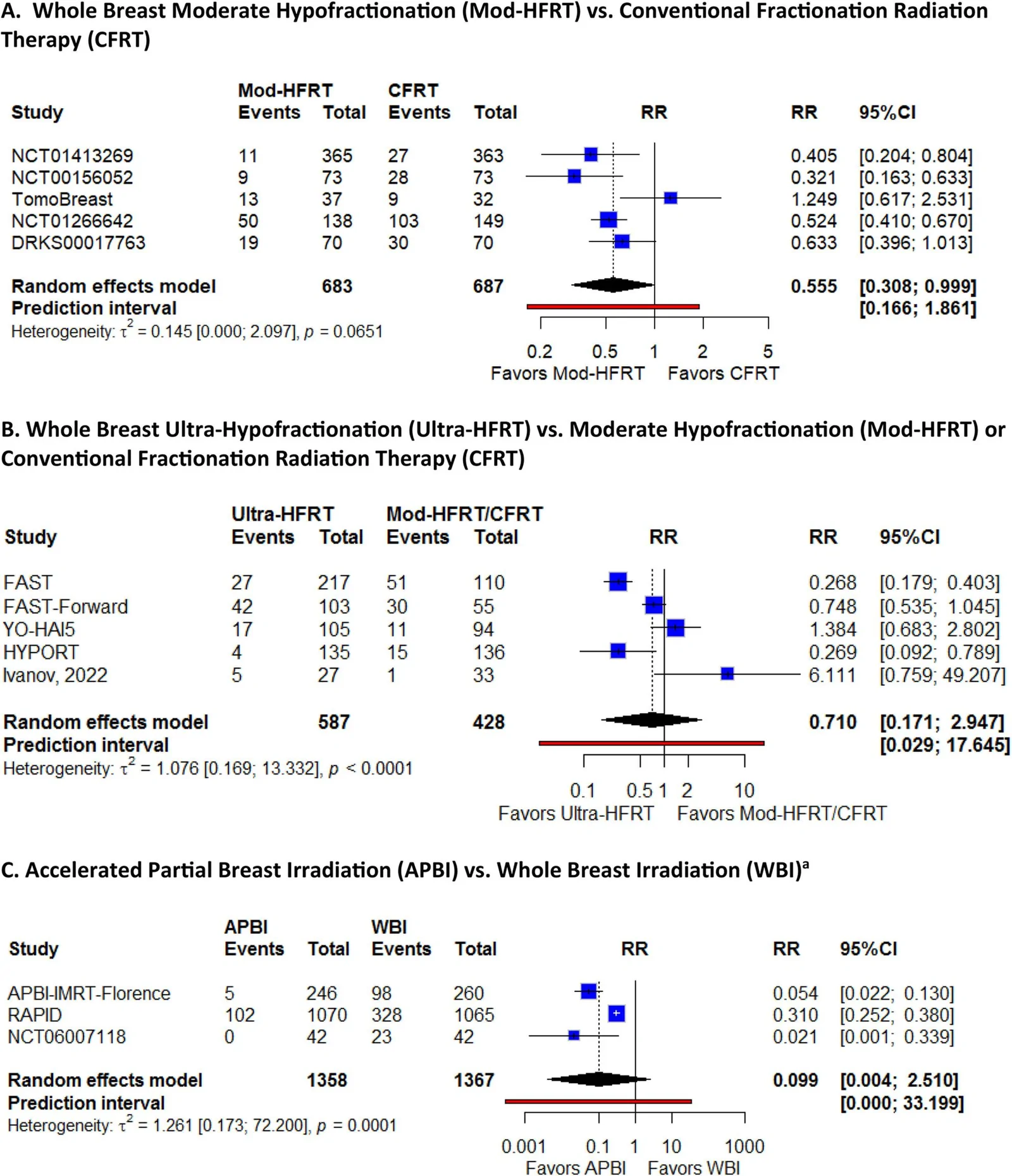
Acute skin toxicity meta-analyses

Moderate hypofractionation may result in little to no difference in acute and late skin toxicity (both low COE; Table 3). Five trials assessed acute skin toxicity over a median follow-up of 4–8 weeks, using CTCAE or RTOG/EORTC criteria [28, 32, 35, 39, 45]. Three trials included breast cancer stages I–II [28, 32, 45] and 2 trials included stages I–III [35, 39]. Pooled analyses across these studies showed RR for acute toxicity of 0.56 (95% CI 0.31, 1.00; *N* = 1,370; Fig. 3A). Two of these studies also reported late skin toxicity, both assessed using RTOG/EORTC criteria with outcomes at 5–10 years [33, 37]. Another study used CTCAE criteria and reported skin toxicity at 3 years [29]. All three studies found no differences between groups, and rates of late skin toxicity were generally low. For example, grade 2–3 skin toxicity at 10 years was 9% (21/235) in the moderate hypofractionation group and 8% (17/220) in the conventional radiation group [33].

Moderate hypofractionation also results in little to no difference in acute pneumonitis, compared with conventional radiotherapy (high COE; Table 3). Two trials (*N* = 1,549) evaluated acute pneumonitis, both using CTCAE criteria [37, 39]. Rates of grade 2 acute pneumonitis were low in both moderate hypofractionation and conventional radiation groups, ranging 2–3% in the former. No grade 3 events were observed in either trial. Only one trial [28] reported results on late pneumonitis, finding no events of any grade in either arm; therefore, we were unable to assess COE for this outcome.

None of the trials reporting toxicity outcomes evaluated for subgroup effects.

#### Quality of life

Three trials evaluated quality of life over follow-up periods of 2–3 years [28, 29, 31, 46]. These trials used the EORTC Quality of Life Questionnaire (QLQ-C30), and/or the Functional Assessment of Cancer Therapy General (FACT-G) and for Breast Cancer (FACT-B) [28, 29]. None of these trials found differences in overall quality of life or global health status between moderate hypofractionation and conventional radiation treatment. One trial also reported no differences in specific domains of functioning (e.g., physical and emotional functioning) [45].

### Whole breast ultra-hypofractionation vs. moderate hypofractionation or conventional fractionation

Five trials (rated low or some concerns RoB) compared whole breast ultra-hypofractionation (total doses 26.0–30.0 Gy, 5.2–6.0 Gy per fraction) with either moderate hypofractionation (total dose 40.05 Gy, 2.67 Gy per fraction) [14, 15, 17, 18] or conventional radiation (total dose 50.0 Gy, 2.0 Gy per fraction) [20, 48] (Table 2). In all studies, radiation therapy occurred after breast surgery and other concurrent treatments were common (chemotherapy, hormone therapy, and/or anti-HER2 therapy). Three trials focused on early stage breast cancer [15, 18, 20], while the other two also included stage III cancer [17, 49]. The largest trials were FAST [20, 48] (*N* = 713) and FAST-Forward [14, 49] (*N* = 4,096), both conducted in the UK, with follow-up duration 5–10 years. The remaining studies were much smaller (*N* = 60–271) and had follow-up of much shorter duration (2 weeks to 18 months) [15, 17, 18]. Only FAST and FAST-Forward reported survival and recurrence outcomes, while the other studies only described findings on toxicity and harms. Detailed findings for all eligible outcomes are provided in **Supplemental Tables 5** and summarized below.

#### Survival & recurrence outcomes

Ultra-hypofractionation results in little to no difference in overall survival or local-regional recurrence, compared with moderate hypofractionation or conventional radiation (high COE for both outcomes; Table 4). Over 5–10 years, overall survival rates were high in all groups (92–98%) [14, 20] and local-regional recurrence rates were low (2.3–3.2%) [14]. Neither FAST nor FAST-Forward reported disease-free survival, or subgroup analyses for survival or recurrence outcomes.

**Table 4:** Certainty of Evidence for Survival & Toxicity Outcomes: Whole Breast Ultra-Hypofractionation (Ultra-HFRT) vs. Moderate Hypofractionation (Mod-HFRT) or Conventional Fractionation Radiation Therapy (CFRT)

Outcome and MCID^a^	Follow-up No. of Participants (Studies)	Relative Effect (95% CI)	Anticipated Absolute Effects (95% CI)			Certainty	What Happens
			Ultra-HFRT	Mod-HFRT/ CFRT	Difference		
**Overall survival** Risk ratio and absolute effect estimates at 5 years from FAST-Forward trial (14) **Threshold: 5% difference**	3–5 years *N =* 5004 (2 RCTs) (14, 20)	**RR = 0.996** (0.98, 1.01)	**92.9%** (91.3, 94.5)	93.2%	**0.4% fewer** (2.0 fewer to 1.3 more)	⨁⨁⨁⨁ High	Ultra-hypofractionation results in little to no difference in overall survival.
**Local-regional recurrence** Risk ratio and absolute effect estimates at 5 years from FAST-Forward trial (14) **Threshold: 10% difference**	5 years *N =* 4096 (1 RCT) (14)	**RR = 0.74** (0.51, 1.08)	**2.3%** (1.6, 3.4)	3.2%	**0.8% fewer** (1.6 fewer to 0.3 more)	⨁⨁⨁⨁ High	Ultra-hypofractionation results in little to no difference in local-regional recurrence.
**Acute skin toxicity** **(grade ≥ 2)** Absolute effect estimates based on control event rate at 23 weeks from HYPORT trial (17) **Threshold: 10% difference**	≤ 6 months *N* = 1015 (5 RCTs) (14, 15, 17, 18, 20)	**RR = 0.71** (0.17, 2.95)	**7.8%** (1.9, 32.5)	11.0%	**3.2% fewer** (9.1 fewer to 21.5 more)	⨁◯◯◯ Very low^a, b,c^	The evidence is very uncertain on the effect of ultra-hypofractionation on acute skin toxicity.

*Abbreviations*: *CFRT *Conventional fractionation radiation therapy, *CI *Confidence interval, *HFRT *Hypofractionation radiation therapy, *NR *Not reported, *RCT *Randomized controlled trial, *RR *Risk ratio

^a^MCIDs are absolute between-group differences in rates

**GRADE Working Group Certainly of Evidence Ratings**:

High certainty: we are very confident that the true effect lies close to that of the estimate of the effect

Moderate certainty: we are moderately confident in the effect estimate: the true effect is likely to be close to the estimate of the effect, but there is a possibility that it is substantially different

Low certainty: our confidence in the effect estimate is limited: the true effect may be substantially different from the estimate of the effect

Very low certainty: we have very little confidence in the effect estimate: the true effect is likely to be substantially different from the estimate of effect

**Explanations**:

a. Downgraded one level for study limitations (studies with some concerns for risk of bias)

b. Downgraded one level for imprecision (CI crossing clinical threshold in one direction)

c. Downgraded one level for inconsistency (substantial heterogeneity across studies)

#### Toxicity & harms

The evidence is very uncertain on the effect of whole breast ultra-hypofractionation on acute skin toxicity compared with moderate hypofractionation or conventional radiation (very low COE; Table 4). All five trials evaluated acute skin toxicity, using either CTCAE or RTOG/EORTC criteria, and reported widely varying rates (Fig. 3B). Grade ≥ 2 acute skin toxicity rates were 11–49% in the ultra-hypofactionation groups, and 20–69% comparator groups (**Supplemental Table 5**) [15, 17, 18, 20, 49]. The pooled estimate across these studies showed a RR of 0.71 (95% CI 0.17, 2.95; Fig. 3B). In addition to inconsistency between studies, there were also methodological concerns with 2 trials (rated some RoB) and substantial imprecision with wide confidence intervals that contributed to the very low COE (Fig. 3B). One small trial (total *N* = 60) reported late skin toxicity and found no grade ≥ 2 events in either group [18]. No trials reported on overall toxicity or lung toxicity.

#### Quality of life

Only one trial evaluated quality of life, assessed with the EORTC QLQ-C30. The ultra-hypofractionation group had a smaller proportion of participants who had decreased ≥ 10 points in global health status, and physical and social functioning at 2–4 weeks after completion of radiation therapy [15]. For example, there were 15% (16/105) of the ultra-hypofractionation group who had decreased ≥ 10 points in global health status, compared with 32% (30/94) in the moderate hypofractionation group (*p* = 0.005, **Supplemental Table 5**) [15].

### Accelerated partial breast irradiation (APBI) vs. whole breast moderate hypofractionation or conventional radiation

Three trials evaluating APBI were rated some concerns for RoB. APBI regimens varied, with total doses 30–38.5 Gy, 3.85–6.0 Gy per fraction, and use of advanced techniques such as intensity modulation (IMRT), 3-dimensional conformal radiation (3DCRT), and volumetric modulated arc therapy (VMAT; Table 2). Two trials compared APBI with whole breast moderate hypofractionation (total doses 40.0–42.5 Gy, 2.65–2.67 Gy per fraction) [23, 24], and the third trial compared APBI with conventional radiation (total dose 50.0 Gy, 2.0 Gy per fraction) [25]. In all studies, radiation therapy occurred after breast-conserving surgery and other systemic treatments were common (Table 2). All three trials focused on early stage breast cancer, and two also included individuals with DCIS [23, 24]. The largest trial, RAPID, was conducted in 33 sites across Canada, Australia, and New Zealand, and enrolled 2,135 women; median follow-up was 8.6 years [24, 52]. The other two trials were both conducted in Europe and had median follow-up of 3.1 to 10.7 years [23, 25]. All three trials evaluated survival, recurrence, and toxicity outcomes, although one of these has only reported toxicity findings thus far [23]. No trial assessed quality of life. Detailed findings for all eligible outcomes are provided in **Supplemental Table 6**.

#### Survival & recurrence outcomes

APBI probably results in little to no difference in overall survival, disease-free survival, or local-regional recurrence rates, compared with whole breast moderate hypofractionation or conventional radiation (moderate COE for all outcomes; Table 5). In both studies reporting these outcomes, survival rates were high and recurrence rates were low across all arms (**Supplemental Table 6**) [24, 25]. For example, overall survival in RAPID was 93% in the APBI group and 94% in the whole breast radiation group, with HR 1.18 (95% CI 0.84, 1.64) [24]. Local recurrence rates were 3.0% in the APBI arm and 2.8% in the whole breast radiation group, with HR 1.27 (90% CI 0.84, 1.91) [24].

**Table 5:** Certainty of Evidence for Survival & Toxicity Outcomes: Accelerated Partial Breast Irradiation (APBI) vs. Whole Breast Hypofrationated or Conventional Radiation Therapy

Outcome and MCID^a^	Follow-up No. of Participants (Studies)	Relative Effect (95% CI)	Anticipated Absolute Effects (95% CI)			Certainty	What Happens
			APBI	Whole Breast	Difference		
**Overall survival** Risk ratio and absolute effect estimates at 8.6 years from RAPID trial (24) **Threshold: 5% difference**	8.6–10 years *N =* 2655 (2 RCTs) (24, 51)	**RR = 0.99** (0.97, 1.01)	**92.9%** (90.8, 95.0)	94.0%	**1.1% fewer** (3.2 fewer to 1 more)	⨁⨁⨁◯ Moderate^a^	Accelerated partial breast irradiation probably results in little to no difference in overall survival.
**Local-regional recurrence** Risk ratio and absolute effect estimates at 10 years from APBI-IMRT Florence trial (51) **Threshold: 10% difference**	10 years *N =* 520 (1 RCT) (51)	**RR = 1.29** (0.49, 3.40)	**3.5%** (1.3, 9.2)	2.7%	**0.8% more** (1.4 fewer to 6.5 more)	⨁⨁⨁◯ Moderate^a^	Accelerated partial breast irradiation probably results in little to no difference in local-regional recurrence.
**Any acute toxicity** **(grade 2–3)** Risk ratio and absolute effect estimates at ≤ 3 months from RAPID trial (24) **Threshold: 10% difference**	≤ 3 or 6 months *N* = 2641 (2 RCTs) (24, 51)	**RR = 0.62** (0.55, 0.69)	**28.0%** (24.9, 31.5)	45.4%	**17.4% fewer** (20.5 fewer to 14 fewer)	⨁⨁⨁◯ Moderate^a^	Accelerated partial breast irradiation probably results in less overall acute toxicity.
**Any late toxicity** **(grade 2–3)** Risk ratio and absolute effect estimates at 8.6 years from RAPID trial (24) **Threshold: 10% difference**	> 3 or 6 months *N* = 2641 (2 RCTs) (24, 51)	**RR = 2.43** (2.03, 2.89)	**32.3%** (27.1, 38.6)	13.3%	**19.0% more** (13.8 more to 25.2 more)	⨁⨁◯◯ Low^a, b^	Accelerated partial breast irradiation may result in more overall late toxicity.
**Acute skin toxicity** **(grade ≥ 2)** Risk ratio and absolute effect estimates based on control event rates at ≤ 3 months from RAPID trial (24) **Threshold: 10% difference**	≤ 6 months *N* = 2725 (3 RCTs) (23–25)	**RR = 0.10** (0.004, 2.51)	**3.0%** (0.1, 77.3)	30.8%	**27.7% fewer** (30.7 fewer to 46.5 more)	⨁◯◯◯ Very low^a, c^	The evidence is very uncertain on the effect of accelerated partial breast irradiation on acute skin toxicity.
**Late skin toxicity** **(grade ≥ 2)** Absolute effect estimates at 5 years from APBI-IMRT Florence trial (25) **Threshold: 10% difference**	> 6 months or 5 years *N* = 590 (2 RCTs) (23, 25)	Not estimable	**0%**	0.01%	**0.8% fewer**	⨁⨁◯◯ Low^a, d^	Accelerated partial breast irradiation may result in little to no difference in late skin toxicity.
**Acute pneumonitis** **(grade 2–3)** Risk ratio and absolute effect estimates at ≤ 3 months from RAPID trial (24) **Threshold: 10% difference**	≤ 3 months *N* = 2219 (2 RCTs) (23, 24)	**RR = 0.25** (0.053, 1.17)	**0.2%** (0, 0.9)	0.8%	**0.6% fewer** (0.7 fewer, 0.1 more)	⨁⨁⨁◯ Moderate^a^	Accelerated partial breast irradiation probably results in little to no difference in acute pneumonitis

*Abbreviations: APBI *Accelerated partial breast irradiation, *CFRT *Conventional fractionation radiation therapy, *CI *Confidence interval, *HFRT *Hypofractionation radiation therapy, *NR *Not reported, *RCT *Randomized controlled trial, *RR *Risk ratio

^a^MCIDs are absolute between-group differences in rates

**GRADE Working Group Certainly of Evidence Ratings**:

High certainty: we are very confident that the true effect lies close to that of the estimate of the effect

Moderate certainty: we are moderately confident in the effect estimate: the true effect is likely to be close to the estimate of the effect, but there is a possibility that it is substantially different

Low certainty: our confidence in the effect estimate is limited: the true effect may be substantially different from the estimate of the effect

Very low certainty: we have very little confidence in the effect estimate: the true effect is likely to be substantially different from the estimate of effect

**Explanations**:

a. Downgraded one level for study limitations (studies with some concerns for risk of bias)

b. Downgraded one level for inconsistency (substantial heterogeneity across studies)

c. Downgraded two levels for imprecision (CI crossing clinical thresholds in both directions)

d. Downgraded one level for imprecision (zero events observed in APBI arm)

Both trials evaluated for subgroup effects on local recurrence at 8–10 years, and examined a variety of characteristics, including patient age, adjuvant therapy, invasive cancer versus DCIS, and tumor size [24, 25]. No moderation effects were found for any of these factors.

#### Toxicity & harms

APBI probably results in less acute overall toxicity (moderate COE), but it may result in more late overall toxicity, compared with whole breast radiation (low COE; Table 5). Two trials [24, 25] reported on acute and late overall toxicity, and both showed less acute overall toxicity in the APBI group. RAPID used CTCAE criteria and defined acute events being ≤ 3 months (24), whereas APBI-IMRT Florence applied RTOG/EORTC criteria and used 6 months as the threshold for acute events [51]. These trials demonstrated inconsistent findings on late overall toxicity, with RAPID reporting more frequent grade ≥ 2 events in the APBI arm (32.3%) compared with whole breast radiation (13.3%) [24], and APBI-IMRT Florence showing the opposite (0% in APBI arm vs. 2.7% in whole radiation group) [51] (**Supplemental Table 6**). Notably, APBI-IMRT Florence also reported fewer acute grade ≥ 2 events in the APBI arm (2.0%) [51] compared with RAPID (28.0%) [24].

The evidence is very uncertain for acute skin toxicity (very low COE) and APBI may result in little to no difference for late skin toxicity, compared with whole breast radiation (low COE; Table 5). In addition to RAPID and APBI-IMRT Florence, the third trial also evaluated acute skin toxicity. This smaller trial (*N* = 84) used CTCAE criteria and defined acute events as ≤ 3 months [23]. There was wide variation in the rates of acute skin toxicity across these studies, with grade ≥ 2 events ranging 0–10% in the APBI arms, and 31–55% in the whole breast radiation groups. The pooled estimate for RR was 0.10 (95% CI 0.004, 2.51) with significant heterogeneity (Fig. 3C). Two of these studies also reported late skin toxicity (> 3 or 6 months) and both reported no grade ≥ 2 late skin toxicity in the APBI arms [23, 25]. Rates in the whole breast radiation groups varied substantially from 0.01% in APBI-IMRT Florence [25], to 12% in the other trial [23].

APBI probably results in little to no difference in acute pneumonitis, compared with whole breast radiation (moderate COE; Table 5) RAPID found low rates of acute grade ≥ 2 pneumonitis in both groups (0.2% in APBI vs. 0.8% in whole breast radiation) [24]. A second trial also evaluated for acute and late pneumonitis but reported no grade ≥ 2 events in either group [23].

No studies evaluated for subgroup effects in toxicity outcomes.

## Discussion

In this updated review of adjuvant hypofractionated radiation therapy for treatment of breast cancer, we found that all included radiation treatments resulted in high survival and low recurrence, with little to no difference between therapies (Table 6). However, there may be important differences in toxicity outcomes. Specifically, moderate hypofractionation probably results in less overall acute toxicity, compared with conventional fractionation, but there was little to no difference in specific events, such as skin toxicity or pneumonitis. Moderate hypofractionation also probably results in little to no difference in overall late toxicity, compared with conventional fractionation. For the other comparisons, toxicity findings were more limited or inconsistent. No trial on whole breast ultra-hypofractionation reported overall toxicity and the evidence on acute skin toxicity is very uncertain. While APBI probably results in less overall acute toxicity, it may also result in more overall late toxicity, compared with whole breast radiation treatments. The evidence was also either very uncertain or showed little to no difference in specific events such as acute skin toxicity or pneumonitis.

**Table 6:** Summary of key findings for all prioritized outcomes by comparison

Outcome^a^	Follow-up	Total *N* (# Trials)	Certainty	Summary Statement
Moderate Hypofractionation (Mod-HFRT) vs. Conventional Fractionation Radiation Therapy (CFRT)^b^				
Overall survival	5–10 years	9436 (7)	⨁⨁⨁⨁ High	Mod-HFRT results in little to no difference in overall survival.
Disease-free survival	5–10 years	7574 (6)	⨁⨁⨁⨁ High	Mod-HFRT results in little to no difference in disease-free survival.
Local-regional recurrence	5–10 years	7948 (6)	⨁⨁⨁⨁ High	Mod-HFRT results in little to no difference in local-regional recurrence
Any acute toxicity	3 months	287 (1)	⨁⨁⨁◯ Moderate	Mod-HFRT probably results in less overall acute toxicity.
Any late toxicity	6 months	271 (1)	⨁⨁◯◯ Low	Mod-HFRT may result in little to no difference in overall late toxicity.
Acute skin toxicity	3 months	1370 (5)	⨁⨁◯◯ Low	Mod-HFRT may result in little to no difference in acute skin toxicity.
Late skin toxicity	5–10 years	2054 (2)	⨁⨁◯◯ Low	Mod-HFRT may result in little to no difference in late skin toxicity.
Acute pneumonitis	6 months	1549 (2)	⨁⨁⨁⨁ High	Mod-HFRT results in little to no difference in acute pneumonitis.
***Ultra-hypofractionation (Ultra-HFRT) vs. Moderate Hypofractionation (Mod-HFRT) or Conventional Fractionation Radiation Therapy (CFRT)*** ^***b***^				
Overall survival	3–5 years	5004 (2)	⨁⨁⨁⨁ High	Ultra-HFRT results in little to no difference in overall survival.
Local-regional recurrence	5 years	4096 (1)	⨁⨁⨁⨁ High	Ultra-HFRT results in little to no difference in local-regional recurrence.
Acute skin toxicity	≤ 6 months	1015 (5)	⨁◯◯◯ Very low	The evidence is very uncertain on the effect of ultra-HFRT on acute skin toxicity.
***Accelerated Partial Breast Irradiation (APBI) vs. Whole Breast Moderate Hypofractionation or Conventional Fractionation*** ^***b***^				
Overall survival	8.6–10 years	2655 (2)	⨁⨁⨁◯ Moderate	APBI probably results in little to no difference in overall survival.
Local-regional recurrence	10 years	520 (1)	⨁⨁⨁◯ Moderate	APBI probably results in little to no difference in little to no difference in local-regional recurrence.
Any acute toxicity	≤ 3 or 6 months	2641 (2)	⨁⨁⨁◯ Moderate	APBI probably results in less overall acute toxicity.
Any late toxicity	> 3 or 6 months	2641 (2)	⨁⨁◯◯ Low	APBI may result in more overall late toxicity.
Acute skin toxicity	≤ 6 months	2725 (3)	⨁◯◯◯ Very low	The evidence is very uncertain on the effect of APBI on acute skin toxicity.
Late skin toxicity	> 6 months or 5 years	590 (2)	⨁⨁◯◯ Low	APBI may result in little to no difference in late skin toxicity.
Acute pneumonitis	≤ 3 months	2219 (2)	⨁⨁⨁◯ Moderate	APBI probably results in little to no difference in acute pneumonitis.

*Abbreviations*: *APBI *Accelerated partial breast irradiation, *CFRT *Conventional fractionated radiation therapy, *CTCAE *Common Terminology Criteria for Adverse Events, *Mod-HFRT *Moderate hypofractionation radiation therapy, *RTOG/ECOG *Radiation Therapy Oncology Group/European Organisation for Research and Treatment of Cancer, *Ultra-HFRT *Ultra-hypofractionated radiation therapy

^a^All toxicity outcomes were grade ≥ 2 based on CTCAE and/or RTOG/ECOG criteria

^b^Radiation treatment regimen doses by comparison:

Mod-HFRT (total dose 40.0–43.5 Gy [2.65–2.9 Gy per fraction]) vs. CFRT (total dose 50.0 Gy [2.0 Gy per fraction])

Ultra-HFRT (total dose 26.0–30.0 Gy [5.2–6.0 Gy per fraction]) vs. Mod-HFRT (total dose 40.05 Gy, 2.67 Gy per fraction) or CFRT (total dose 50.0 Gy [2.0 Gy per fraction])

APBI (total dose 30–38.5 Gy [3.85–6.0 Gy per fraction]) vs. Whole Breast Mod-HFRT (40.0–42.5 Gy [2.65–2.67 Gy per fraction]) or CFRT (total dose 50.0 Gy [2.0 Gy per fraction])

Our results showing no differences in survival and recurrence for moderate hypofractionation vs. conventional radiation are consistent with past systematic reviews on hypofractionated radiation therapy for breast cancer [54–56], and add further support to current ASTRO recommendations for using moderate hypofractionation over conventional radiation [57]. Since the 2018 ASTRO guidelines were published, additional trials have demonstrated that moderate hypofractionation produces similar effects on survival and recurrence for DCIS [30, 31], and in varied settings and locations around the world [30, 31, 37, 39]. Subgroup analyses have also shown that moderate hypofractionation has similar efficacy across a range of patient and disease characteristics, compared with whole breast radiation. Our results showing that moderate hypofractionation results in less overall acute toxicity, with little to no differences in acute skin toxicity or pneumonitis, also reinforce the desirability of this option as first-line treatment for breast cancer. Recent prior reviews [54–56] did not examine overall toxicity (acute or late), but two of these reviews concluded that moderate hypofractionation resulted in less acute skin toxicity [54] or radiation dermatitis [55]. It is unclear whether differences in our findings are due in part to the inclusion of findings from high RoB trials by these prior reviews, but notably, they also concluded there were no differences in most toxicity outcomes, including other specific acute toxicity (e.g., pneumonitis) or various late adverse events [55]. We also note there is a lack of evidence on whether toxicity outcomes may differ across patient subgroups, when comparing moderate hypofractionation with conventional radiation. Our review also found that whole breast ultra-hypofractionation has similar effects on overall survival and local-regional recurrence, when compared with moderate hypofractionation or conventional radiation. While prior systematic reviews on hypofractionation often examined ultra-hypofractionation in the same analyses with moderate hypofractionation [7, 58, 59], a recently completed review also separated out ultra-hypofractionation trials for separate analyses [55]. This review similarly concluded that there are no differences in survival and recurrence [55]. Regarding toxicity, we did not identify any studies reporting overall toxicity; for specific toxicity, the evidence was either very uncertain or low certainty. The prior review separately examining ultra-hypofractionation also evaluated a variety of specific toxicity effects, finding there may be no differences in acute dermatitis but ultra-hypofractionation increased rates for some harms (e.g., breast or chest wall induration/fibrosis) [55]. Before future implementation of whole breast ultra-fractionation should be recommended, more work is needed to understand differences in toxicity, particularly for long-term outcomes compared with moderate hypofractionation. Given the lack of studies reporting on subgroup effects, it will also be important for future research to examine whether effects on survival, recurrence, and toxicity may vary by patient and/or disease characteristics.

Compared to prior systematic reviews on APBI [60, 61], we identified additional trials [13, 22, 23], and excluded studies using brachytherapy or intra-operative radiation therapy. However, our findings on survival, recurrence and toxicity for APBI vs. whole breast radiation are largely consistent with results from past reviews [60, 61]. One of the newer trials included in our review provided more data regarding specific toxicity, such as pneumonitis or lymphedema, but it did not report overall toxicity [23]. We also found a large French trial (SHARE) [13] that published its results recently but it was rated high RoB due to the great extent of non-adherence to treatment assignment (e.g., 20% of participants assigned to APBI received radiation treatments outside of protocol). Future research with longer follow-up is needed to confirm whether APBI does indeed result in higher overall late toxicity, as this would substantially limit its use. Given lack of evidence on subgroup effects, studies should also consider whether toxicity may vary across important patient characteristics. Additionally, current evidence on these radiation treatments largely comes from Europe and North America; it will be important to examine whether survival and recurrence outcomes will be similar across other settings and locations around the world.

The push to develop and evaluate whole breast ultra-hypofractionation and APBI regimens reflects the importance of reducing treatment burden and toxicity of radiation treatments, particularly for those with early stage breast cancer, who generally have high survival and low recurrence rates. There is also hope that such newer radiation therapies may improve access to care, since these treatments have shorter durations. However, the sophisticated technology and additional planning needed for the closely controlled delivery of complex radiation treatments may also be resource intensive and inaccessible in certain communities or settings. In future evaluations of whole breast ultra-hypofractionation and APBI, it will be critical to examine implementation costs and barriers, and whether these may vary across different settings.

This review has several limitations. We focused on hypofractionated or conventional radiation therapy with curative intent for breast cancer. Studies evaluating other techniques such as brachytherapy or use of radiation for palliative intent were excluded. We also did not include observational studies because RCTs provide the highest level of evidence particularly for clinical efficacy. However, rare serious adverse events are not likely to be detected in RCTs with even a few thousand participants. Additionally, we only abstracted detailed findings from RCTs rated low or some concerns for RoB to focus on higher quality evidence. Eligible studies were also limited to publications in English; there may have been relevant trials published in other languages that were not included.

## Conclusions

In conclusion, whole breast hypofractionation or conventional radiation and APBI have similar effects on survival and recurrence, particularly for early stage breast cancer. Moderate hypofractionation and APBI result in lower overall acute toxicity, but the evidence is very uncertain for whole breast ultra-hypofractionation. For both whole breast ultra-hypofractionation and APBI, future research is also needed to better understand late toxicity outcomes, and whether effects may vary across settings and subgroups.

## Supplementary Information

Supplementary Material 1.

## Data Availability

All data generated or analyzed during this study are included in this published article and its supplementary information files.

## Abbreviations

3DCRT: 3-dimensional conformal radiation
ASTRO: American Society for Radiation Oncology
APBI: Accelerated partial breast irradiation
CFRT: Conventionally fractionated radiation therapy
CI: Confidence interval
COE: Certainty of evidence
CTCAE: Common Terminology Criteria for Adverse Events
DCIS: Ductal carcinoma in situ
EORTC: European Organization for Research and Treatment of Cancer
ESP: Evidence Synthesis Program
FACT-B: Functional Assessment of Cancer Therapy for Breast Cancer
FACT-G: Functional Assessment of Cancer Therapy General
IMRT: Intensity modulation radiation therapy
GRADE: Grading of Recommendations, Assessment, Development and Evaluation
HR: Hazard ratio
KQ: Key question
QLQ-C30: Quality of Life Questionnaire
RCT: Randomized controlled trial
RoB: Risk of bias
RoB 2: Cochrane Risk of Bias for Randomized Trials 2
RR: Relative risk
RTOG: Radiation Therapy Oncology Group
START: Standardisation of Breast Radiotherapy
UK: United Kingdom
VA: US Department of Veterans Affairs
VHA: US Veterans Health Administration
VMAT: Volumetric modulated arc therapy
