# Supplementary material for: Comparative efficacy and harms of adjuvant whole breast hypofractionation or external beam accelerated partial breast irradiation for treatment of breast cancer: a systematic review
Source: BMC Cancer. 2026 Jun 25;26:1102. doi: 10.1186/s12885-026-16129-5 (PMC13579765; doi:10.1186/s12885-026-16129-5)
Supplement: Supplementary file 1 [file 12885_2026_16129_MOESM1_ESM.docx]

**Contents**

[Supplemental Table 1. Search Strategies	1](#_Toc193733561)

[Supplemental Table 2. Eligibility Criteria for Selection of Relevant Studies.	2](#_Toc193733562)

[Supplemental Table 3. Risk of Bias Ratings for All Eligible Breast Cancer Trials	4](#_Toc193733563)

[Supplemental Table 4. Detailed Results for Survival and Recurrence Outcomes for Trials Rated “Low” or “Some Concerns” Risk of Bias	7](#_Toc193733564)

[Supplemental Table 5. Detailed Results for Harms Outcomes for Trials Rated “Low” or “Some Concerns” Risk of Bias	14](#_Toc193733565)

[Supplemental Table 6. Detailed Results for Quality of Life Outcomes for Breast Cancer Trials Rated “Low” or “Some Concerns” Risk of Bias	20](#_Toc193733566)

[Supplemental Material 1: PRISMA 2020 Checklist	22](#_Toc193733567)

[References	26](#_Toc193733568)

**Supplemental Table 1**. Search Strategies

|  | **Search Terms (OVID MEDLINE and Embase)^a^** |
| --- | --- |
| 1 | exp Adenocarcinoma/ or adenocarcinoma.mp. |
| 2 | neoplasm.mp. or exp Neoplasms/ |
| 3 | (hematologic or haematologic or lymphoma or leukemia).ti,ab. |
| 4 | 1 or 2 |
| 5 | 4 not 3 |
| 6 | Radiation Dose Hypofractionation/ or (radiotherapy minibeam$1 or radiation hypofractionated dose or radiation dose hypofractionation or hypofractionated radiation therapy or short?course radiation therapy).ti,ab. |
| 7 | dose fractionation/ or dose response relationship, radiation/ or radiotherapy dosage/ |
| 8 | ((radiotherapy* or radiat*) adj2 (dose or dosage or regimen* or schedule*)).tw. |
| 9 | hypofractionat*.mp. |
| 10 | hypo-fraction*.mp. |
| 11 | multi-fraction*.tw. |
| 12 | (hypo adj3 fraction*).tw. |
| 13 | Stereotactic body radiation therapy/ or SBRT.mp. |
| 14 | Stereotactic ablative body radiation therapy/ or SABR.mp. |
| 15 | (Stereotactic body radiation therapy or SBRT).tw. |
| 16 | (Stereotactic ablative body radiation therapy or SABR).tw. |
| 17 | or/6-16 |
| 18 | 5 and 17 |
| 19 | Randomized controlled trial.pt. or randomized.mp. or placebo.mp. |
| ‘21 | ('clinical ’trial‘ or 'randomized controlled ’study‘ or 'randomized controlled ’rial‘ or 'double blind clinical ’study‘ or 'single blind clinical ’tudy‘ or 'random alloc’tion').ti,ab. |
| 22 | (meta-analy$ or metaanaly$ or meta analy$).tw. or exp Meta-Analysis/ or (systematic adj (review$ or overview$)).tw. or (systematic review or literature review or rapid review or umbrella review or meta synthesis or metasynthesis or meta-analysis or meta-synthesis or integrative review or data synthesis or comparative effectiveness review).mp |
| 23 | or/19-22 |
| 24 | (Animals/ or Models, Animal/ or Disease Models, Animal/) not Humans/ |
| 25 | ((animal or animals or canine* or cat or cats or dog or dogs or feline or goat or hamster* or horse or lamb or lambs or mice or monkey or monkeys or mouse or murine or pig or pigs or piglet* or porcine or primate* or rabbit* or rats or rat or rodent* or sheepurrentryrinar*) not (human* or patient*)).ti,kf,jw. |
| 26 | 24 or 25 |
| 27 | 23 not 26 |
| 28 | 18 and 27 |
| 29 | limit 28 to (case reports or comment or editorial or letter or news or newspaper article or personal narrative or conference abstract) [Limit not valid in Embase; records were retained] |
| 30 | 28 not 29 |
| 31 | limit 3 to English language |
| 32 | (child or children or pediat* or neonat*).ti,ab. |
| 33 | 31 not 32 |
| *^a^Original search conducted on 1/5/22, and updated for breast cancer trials on 3/12/24. Additional processing of search results in EndNote included:*   - *Removed duplicates* - *Removed articles containing words in abstract/title: “childhood”, “commentary”, “abstract”, “annual meeting”, “conference”, “proceedings”, or “symposium”*   *These results were then uploaded into DistillerSR and further de-duplicated.* | |

Supplemental Table 2. Eligibility Criteria for Selection of Relevant Studies.

| **Eligibility Criteria** | |
| --- | --- |
| **Population** | Adults, 18 years or older, diagnosed with any of the following cancers^a^: breast, prostate, lung, rectal, head and neck, bladder, pancreas, melanoma, and non-melanoma skin cancer receiving radiation with definitive treatment intent (*ie*, non-palliative) |
| **Intervention** | Hypofractionation (>2.2 Gy per fraction)   - Moderate hypofractionation - Ultrahypofractionation/extreme hypofractionation - Stereotactic body radiation therapy (SBRT)/Stereotactic ablative body radiation therapy (SABR) |
| **Comparator** | Conventional radiation (1.8 – 2.2 Gy per fraction) or other standard of care radiation therapy |
| **Outcomes** | Survival: Overall, Disease-specific  Recurrence: Local, Regional, Systemic/distant metastasis  Toxicity: All adverse events of grade 2-5, specific adverse events grade 2-5 relevant to each cancer  Quality of Life: Overall and cancer-specific  Cost/resource use |
| **Timing** | Effectiveness outcomes timing: short-term (≤2 years) vs long-term (>2 years)  Toxicity timing: any, acute (≤ 90 days post treatment), late (> 90 days post treatment) |
| **Setting** | Any non-hospice setting |
| **Study Design** | Randomized controlled trials |

*^a^Original VA ESP report addressed all of the cancers listed here. Only findings related to breast cancer treatment are included in this paper.*

Supplemental Table 3. Risk of Bias Ratings for All Eligible Breast Cancer Trials

| **Trial** | **Outcome** | **Domain 1: Risk of Bias Arising from the Randomization Process** | **Domain 2a: Risk of Bias Due to Deviations from the Intended Interventions (Effect of Assignment to Intervention)** | **Domain 2b: Risk of Bias Due to Deviations from the Intended Interventions (Effect of Adherence to Intervention)** | **Domain 3: Risk of Bias Due to Missing Outcome Data** | **Domain 4: Risk of Bias in Measurement of Outcome** | **Domain 5: Risk of Bias in Selection of the Reported Result** | **Overall Risk of Bias** |
| --- | --- | --- | --- | --- | --- | --- | --- | --- |
| APBI-IMRT-Florence (1-3) | Mortality | Low | Low | Some concerns | Low | Low | Low | Some concerns |
|  | Survival | Low | Low | Some concerns | Low | Low | Low | Some concerns |
|  | Harms | Low | Low | Some concerns | Low | Low | Low | Some concerns |
| Baillet (4) | Harms | Some concerns | Some concerns | Some concerns | Low | Low | Some concerns | High |
| Das (5) | Survival | Some concerns | Some concerns | High | High | Low | Some concerns | High |
|  | Harms | Some concerns | Some concerns | High | High | Low | Some concerns | High |
| FAST (6, 7) | Mortality (6) | Low | Low | Low | Low | Low | Low | Low |
|  | Survival (6, 7) | Low | Low | Low | Low | Low | Low | Low |
|  | Harms (6) | Low | Low | Low | Low | Low | Low | Low |
| FAST-Forward (8, 9) | Mortality (8) | Low | Low | Low | Low | Low | Low | Low |
|  | Survival (8) | Low | Low | Low | Low | Low | Low | Low |
|  | Harms (9) | Low | Low | Low | Some concerns | Low | Low | Some concerns |
| Hosseini (10) | Harms | Some concerns | Low | Low | Some concerns | Low | Some concerns | High |
| Hou (11) | Mortality | Some concerns | Low | Low | Low | Low | Low | High |
|  | Survival | Some concerns | Low | Low | Low | Low | Low | High |
| HYPORT (12) | Mortality | Low | Low | Low | Low | Low | Low | Low |
|  | Survival | Low | Low | Low | Low | Low | Low | Low |
|  | Harms | Low | Low | Low | Low | Low | Low | Low |
|  | QoL | Low | Low | Low | Low | Low | Low | Low |
| Ivanov, 2022 (13) | Harms | Some concerns | Low | Low | Low | Low | Some concerns | Some concerns |
| Kalita (14) | Harms | Some concerns | Some concerns | High | Low | Low | Some concerns | High |
| King (15) | QoL | Low | Low | Low | Low | Some concerns | Low | Some concerns |
| Kumbhaj (16) | Survival | Some concerns | Some concerns | High | High | Low | Some concerns | High |
|  | Harms | Some concerns | Some concerns | High | High | High | Some concerns | High |
| Maiti (17) | Mortality | High | High | High | Low | Low | Low | High |
|  | Survival | High | High | High | Some concerns | Low | Low | High |
|  | Harms | High | High | High | Some concerns | Low | Low | High |
| NCT00156052 (18-20) | Mortality (19, 20) | Low | Low | Low | Low | Low | Low | Low |
|  | Survival (19, 20) | Low | Low | Low | Low | Low | Low | Low |
|  | Harms (18, 19) | Low | Low | Low | Some concerns | Low | Low | Some concerns |
|  | QoL (18) | Low | Low | Some concerns | High | High | Low | High |
| NCT01266642 (21-23) | Survival (23) | Some concerns | Low | Low | Some concerns | Low | Some concerns | High |
|  | Harms (22, 23) | Some concerns | Low | Low | Low | Low | Low | Some concerns |
|  | QoL (21-23) | Some concerns | Low | Low | Some concerns | Low | Low | Some concerns |
| NCT03324802 (24) | Mortality | Some concerns | Low | Low | High | Low | Low | High |
|  | Survival | Some concerns | Low | Low | High | Low | Low | High |
|  | Harms | Some concerns | Low | Low | High | Low | Low | High |
|  | QoL | Some concerns | Low | Low | High | Low | Low | High |
| NCT06007118 (25) | Mortality | Some concerns | Low | Low | Low | Low | Low | Some concerns |
|  | Survival | Some concerns | Low | Low | Low | Low | Low | Some concerns |
|  | Harms | Some concerns | Low | Low | Low | Low | Low | Some concerns |
|  | QoL | Some concerns | Low | Low | Low | Low | Low | Some concerns |
| NCT40155531 (26) | Harms | Some concerns | Low | Some concerns | High | Low | Low | High |
|  | QoL | Some concerns | Low | Some concerns | High | Low | Low | High |
| Offersen (27) | Mortality | Low | Low | Low | Low | Low | Low | Low |
|  | Survival | Low | Low | Low | Low | Low | Low | Low |
| Owen (28) | Survival | Low | Some concerns | Some concerns | Low | Low | Low | Some concerns |
| RAPID (29, 30) | Mortality (30) | Low | Low | Low | Low | Low | Low | Low |
|  | Survival (30) | Low | Low | Low | Low | Low | Low | Low |
|  | Harms (29, 30) | Low | Low | Low | Some concerns | Low | Low | Some concerns |
| Purohit (31) | Harms | Some concerns | Some concerns | High | High | Some concerns | Some concerns | High |
| Rastogi (32) | Survival | Some concerns | Low | Some concerns | High | Some concerns | Some concerns | High |
|  | Harms | Some concerns | Low | Some concerns | High | Some concerns | Some concerns | High |
| Rodriguez-Li (33, 34) | Mortality (33) | Some concerns | Some concerns | High | Low | Low | Low | High |
|  | Survival (33, 34) | Some concerns | Some concerns | High | Low | Low | Low | High |
|  | Harms (33, 34) | Some concerns | Some concerns | High | Some concerns | Low | Low | High |
| Schmeel (35) | Harms | Some concerns | Low | Some concerns | Low | Low | Low | Some concerns |
| Shahid (36) | Mortality | Some concerns | Some concerns | High | Some concerns | Low | Low | High |
|  | Survival | Some concerns | Some concerns | High | Some concerns | Low | Low | High |
|  | Harms | Some concerns | Some concerns | High | Some concerns | Low | Low | High |
| SHARE (37) | Mortality | Some concerns | Low | Some concerns | High | Some concerns | Low | High |
|  | Survival | Some concerns | Low | Some concerns | High | Some concerns | Low | High |
|  | Harms | Some concerns | Low | Some concerns | High | Some concerns | Low | High |
|  | QoL | Some concerns | Low | Some concerns | High | Some concerns | Low | High |
| Spooner (38) | Mortality | Some concerns | Low | Some concerns | Low | Low | Low | Some concerns |
|  | Survival | Some concerns | Low | Some concerns | Low | Low | Low | Some concerns |
| START (39-41) | Mortality | Low | Low | Low | Low | Low | Low | Low |
|  | Survival | Low | Low | Low | Low | Low | Low | Low |
| Swanick (42) | QoL | Some concerns | Low | Low | Some concerns | Some concerns | Low | High |
| Taher (43) | Harms | High | Low | High | Low | Some concerns | Some concerns | High |
| TomoBreast (44, 45) | Harms (45) | Some concerns | Low | Some concerns | Low | Low | Low | Some concerns |
|  | QoL (44) | Some concerns | Low | Some concerns | Low | Low | Low | Some concerns |
| Van Hulle (46) | Harms | Some concerns | Some concerns | Some concerns | Low | Low | Low | Some concerns |
|  | QoL | Some concerns | Some concerns | Some concerns | Low | Low | Low | Some concerns |
| Wang 2019 (47) | Mortality | Low | Low | Low | Low | Low | Low | Low |
|  | Survival | Low | Low | Low | Low | Low | Low | Low |
|  | Harms | Low | Low | Low | Low | Low | Low | Low |
| Wang 2020 (48) | Mortality | Low | Low | Low | Low | Low | Low | Low |
|  | Survival | Low | Low | Low | Low | Low | Low | Low |
|  | Harms | Low | Low | Low | Low | Low | Low | Low |
| Yadav (49) | Harms | High | Some concerns | High | Low | Low | Low | High |
| Zhao 2016 (50) | Mortality | Some concerns | Some concerns | Some concerns | Some concerns | Low | Low | High |
|  | Survival | Some concerns | Some concerns | Some concerns | Some concerns | Low | Low | High |
|  | Harms | Some concerns | Some concerns | Some concerns | Some concerns | Low | Low | High |
| Zhao 2017 (51) | Mortality | Some concerns | Some concerns | High | Low | Low | Low | High |
|  | Survival | Some concerns | Some concerns | High | Low | Low | Low | High |
|  | Harms | Some concerns | Some concerns | High | Low | Low | Low | High |

Supplemental Table 4. Detailed Results for Moderate Hypofractionation vs. Conventional Radiation Trials (Rated “Low” or “Some Concerns” Risk of Bias)

| **Outcome** | **Trial Name, Year**  **Trial #**  **Risk of Bias** | **Effect Measure/Definition** | **Intervention Arm**  **% (N Events/Total N)** | **Comparison Arm**  **% (N Events/Total N)** | **Results** |
| --- | --- | --- | --- | --- | --- |
| *Survival & Recurrence* | | | | | |
| Overall survival | **DBCG HYPO**  NCT00909818  (Offersen, 2020) (27)  LOW | 9-year survival | 93% (deaths: 60/917) | 93% (deaths: 61/937) | HR (95% CI): 0.98 (0.65, 1.47)  RD (95% CI): 0.0% (−2.9%, 2.8%), P = 0.93 |
|  | NCT00156052  (Whelan, 2002 (20), 2010 (19))  LOW | 10 years | 84.6% (deaths: 122/622) | 84.4% (deaths: 126/612) | RD (95% CI): -0.2% (-4.3%, 4.0%), P = 0.79 |
|  |  | 5 years (median follow-up 69 months) | 92.3% (deaths: 48/622) | 91.7% (deaths: 51/612) | P = 0.78 |
|  | NCT00793962  (Wang, 2019) (47)  LOW | Deaths all-cause, median follow-up 59.5 months | 84% (deaths: 63/401) | 86% (deaths: 56/409) | HR (95% CI): 1.13 (0.78, 1.62)  Log-rank P = 0.53 |
|  | NCT01413269  (Wang, 2020) (48)  LOW | Death from any cause at 5 years (median follow-up 73.5 months) | 97.5% (deaths: 11/365) | 98% (deaths: 9/364) | HR (95% CI): 1.20 (90.50, 2.80)  Log-rank P = 0.680 |
|  | **START A**  (START Trialists, 2008 (39); Haviland, 2013 (41))  LOW | Median follow-up 5.1 and 9.3 years | Arm A (41.6 Gy):  5 years: 88% (deaths: 89/750)  9 years: 83% (deaths: 128/750)    Arm B (39 Gy):  5 years: 89% (deaths: 83/737)  9 years: 82% (deaths: 134/737) | 5 years: 89% (deaths: 84/749)  9 years: 83% (deaths: 130/749) | HR (95% CI):  Arm A (41.6 Gy): 1.04 (0.77, 1.40), P = 0.81  Arm B (39 Gy): 1.00 (0.74, 1.36), P = 0.99 |
|  | **START B**  ISRCTN59368779  (START Trialists, 2008 (40); Haviland, 2013 (41))  LOW | Median follow-up 6 and 9.9 years | 6 years: 90% (deaths: 107/1110)  10 years: 86% (deaths: 159/1110) | 6 years: 88% (deaths: 138/1105)  10 years: 83% (deaths: 192/1105) | HR (95% CI): 0.80 (0.65, 0.99), P = 0.04 |
|  | **TomoBreast**  NCT00459628  (Van Parijs, 2023 (52))  SOME CONCERNS | Deaths at 10 years | 86.4% | 89.1% | P = 0.829 |
|  | **Trial Name/# NR**  (Spooner, 2012) (38)  SOME CONCERNS | Deaths at 2, 5, 10, 15 years | 2 years: 94% (deaths: 11/181)  5 years: 85% (deaths: 27/181)  10 years: 70% (deaths: 54/181)  15 years: 53% (deaths: 85/181) | 2 years: 92% (deaths: 7/177)  5 years: 81% (deaths: 34/177)  10 years: 67% (deaths: 58/177)  15 years: 52% (deaths: 85/177) | HR (95% CI): 1.02 (0.76, 1.35) |
| Disease-free survival | NCT00156052  (Whelan, 2002) (20)  LOW | Free from events (local, regional, and distant recurrence; deaths) within 5 years (median follow-up 69 months) | 85.4% (events: 91/622) | 87.1% (events: 79/612) | P = 0.37 |
|  | NCT00793962  (Wang, 2019) (47)  LOW | Free from locoregional recurrence, distant metastasis, or death, median follow-up 59.5 months | 76% (events: 96/401) | 73% (events: 109/401) | HR (95% CI): 0.88 (0.67, 1.16)  Log-rank P = 0.43 |
|  | NCT01413269  (Wang, 2020) (48)  LOW | 5-year survival from events (local or locoregional recurrence, distant metastasis, or death due to any cause) | 93% (events: 32/365) | 94% (events: 26/364) | HR (95% CI): 1.24 (0.74, 2.07)  Log-rank P = 0.421 |
|  | **START A**  ISRCTN59368779  (START Trialists, 2008 (39); Haviland, 2013 (41))  LOW | Survival from any breast cancer-related event including local, regional, or distant relapse, breast cancer death, or contralateral breast cancer, median follow-up 5.1 and 9.3 years | Arm A (41.6 Gy):  5 years: 88% (events: 91/750)  9 years: 80% (events: 149/750)    Arm B (39 Gy):  5 years: 84% (events: 115/737)  9 years: 78% (events: 163/737) | 5 years: 86% (events: 102/749)  9 years: 79% (events: 154/749) | HR (95% CI):  Arm A (41.6 Gy): 0.94 (0.75, 1.17), P = 0.57  Arm B (39 Gy): 1.08 (0.87, 1.35), P = 0.48 |
|  | **START B**  ISRCTN59368779  (START Trialists, 2008 (40); Haviland, 2013 (41))  LOW | Survival from any breast cancer-related event including local, regional, or distant relapse, breast cancer death, or contralateral breast cancer, median follow-up 6.0 and 9.9 years | 6 years: 89% (events: 127/1110)  10 years: 84% (events: 182/1110) | 6 years: 85% (events: 164/1105)  10 years: 80% (events: 222/1105) | HR (95% CI): 0.79 (0.65, 0.97), P = 0.02 |
|  | **TomoBreast**  NCT00459628  (Van Parijs, 2023 (52))  SOME CONCERNS | Any recurrence, new primary, or death at 10 years | 78.0% | 75.0% | P = 0.256 |
|  | **Trial Name/# NR**  (Spooner, 2012) (38)  SOME CONCERNS | Any recurrence or death at 2, 5, 10, 15 years | 2 years: 89% (events: 20/181)  5 years: 81% (events: 34/181)  10 years: 61% (events: 67/181)  15 years: 46% (events: 98/181) | 2 years: 86% (events: 25/177)  5 years: 73% (events: 48/177)  10 years: 59% (events: 73/177)  15 years: 44% (events: 99/177) | HR (95% CI): 0.98 (0.75, 1.29) |
| Local recurrence | NCT00156052  (Whelan, 2002, (20) Whelan, 2010, (19) Goldberg, 2023 (53))  LOW | Recurrent  tumor within the treated breast within 5 years (median follow-up 69 months) | 2.8% (events: 21/622) | 3.2% (events: 23/612) | RD (95% CI): 0.4%  (-1.5%, 2.4%)  P-value NR |
|  |  | Recurrent  tumor within the treated breast within 10 years | 6.2% (events: 41/622) | 6.7% (events: 42/612) | RD (95% CI): 0.5%  (-2.5%, 3.5%)  Noninferiority test P < 0.001  HR (95% CI): 1.01 (0.67-1.52), P = 0.95 |
|  | NCT01413269  (Wang, 2020) (48)  LOW | 5-year relapse in breast or chest wall | 1% (events: 5/365) | 2% (events: 8/364) | HR (90% CI): 1.63 (0.64, 4.15)  Noninferiority test P = 0.017 |
|  | **START A**  ISRCTN59368779  (START Trialists, 2008 (39); Haviland, 2013 (41))  LOW | Recurrence in breast or chest wall, median follow-up 5.1 and 9.3 years | Arm A (41.6 Gy):  5 years: 4% (events: 28/750)  9 years: 5% (events: 37/750)    Arm B (39 Gy):  5 years: 4% (events: 31/737)  9 years: 6% (events: 47/737) | 5 years: 3% (events: 25/749)  9 years: 5% (events: 40/749) | HR (95% CI):  Arm A (41.6 Gy): 0.90 (0.57, 1.40), P = 0.63  Arm B (39 Gy): 1.20 (0.79, 1.83), P = 0.39 |
|  | **START B**  ISRCTN59368779  (START Trialists, 2008 (40); Haviland, 2013 (41))  LOW | Recurrence in breast or chest wall, median follow-up 6.0 and 9.9 years | 6 years: 2% (events: 25/1110)  10 years: 3% (events: 36/1110) | 6 years: 3% (events: 34/1105)  10 years: 5% (events: 50/1105) | HR (95% CI): 0.70 (0.46, 1.07), P = 0.10 |
|  | **START Pilot**  Trial # NR  (Owen, 2006) (28)  SOME CONCERNS | 10-year recurrence (ipsilateral breast or overlying skin) | 42.9 Gy: 9% (events: 42/466)  39 Gy: 14% (events: 66/474) | 11% (events: 50/470) | HR (95% CI):  42.9 Gy: 0.86 (0.57, 1.30)  39 Gy: 1.33 (0.91, 1.92) |
| Locoregional recurrence | **DBCG HYPO**  NCT00909818  (Offersen, 2020) (27)  LOW | 9-year recurrence (ipsilateral recurrence in the breast tissue and overlying skin, in ipsilateral axilla, fossa supra-clavicularis, or in the internal mammary chain lymph nodes) | 2% (events: 14/794) | 2% (events: 19/814) | HR (95% CI): 0.90 (0.51, 1.59)  RD (95% CI ): −0.3% (−2.3%, 1.7%)  P-value NR |
|  | NCT00793962  (Wang, 2019) (47)  LOW | Recurrence in ipsilateral chest wall or regional lymph nodes, median follow-up 59.5 months | 8% (events: 31/401) | 9% (events: 29/401) | HR (90% CI): 1.10 (0.72, 1.69)  Non-inferiority P < 0.0001 |
|  | NCT01413269  (Wang, 2020) (48)  LOW | 5-year disease recurrence in the ipsilateral  breast and/or regional lymph nodes | 3% (events: 14/365) | 4% (events: 12/364) | HR (95% CI): 0.87 (0.46, 1.66)  Log-rank P = 0.758 |
|  | **START A**  ISRCTN59368779  (START Trialists, 2008 (39); Haviland, 2013 (41))  LOW | Local or ipsilateral  axilla, or supraclavicular fossa, median follow-up 5.1 and 9.3 years | Arm A (41.6 Gy):  5 years: 4% (events: 30/750)  9 years: 6% (events: 42/750)    Arm B (39 Gy):  5 years: 5% (events: 35/737)  9 years: 7% (events: 52/737) | 5 years: 4% (events: 28/749)  9 years: 6% (events: 45/749) | HR (95% CI):  Arm A (41.6 Gy): 0.91 (0.59, 1.38), P = 0.65  Arm B (39 Gy): 1.18 (0.79, 1.76), P = 0.41 |
|  | **START B**  ISRCTN59368779  (START Trialists, 2008 (40); Haviland, 2013 (41))  LOW | Local or ipsilateral  axilla, or supraclavicular fossa, median follow-up 6.0 and 9.9 years | 6 years: 3% (events: 29/1110)  10 years: 4% (events: 42/1110) | 6 years: 3% (events: 36/1105)  10 years: 5% (events: 53/1105) | HR (95% CI): 0.77 (0.51, 1.16), P = 0.21 |
|  | **Trial Name/# NR**  (Spooner, 2012) (38)  SOME CONCERNS | 5-year recurrence | 43% (events: 25/181) | 40% (events: 21/177) | HR NR (“no significant differences”) |
| Distant metastasis | **START A**  ISRCTN59368779  (START Trialists, 2008 (39); Haviland, 2013 (41))  LOW | Relapse in non-irradiated organs, median follow-up 5.1 and 9.3 years | Arm A (41.6 Gy):  5 years: 9% (events: 69/750)  9 years: 15% (events: 110/750)    Arm B (39 Gy):  5 years: 13% (events: 93/737)  9 years: 16% (events: 121/737) | 5 years: 10% (events: 73/749)  9 years: 13% (events: 100/749) | HR (95% CI):  Arm A (41.6 Gy): 1.08 (0.82, 1.41), P = 0.58  Arm B (39 Gy): 1.24 (0.95, 1.61), P = 0.11 |
|  | **START B**  ISRCTN59368779  (START Trialists, 2008 (40); Haviland, 2013 (41))  LOW | Relapse in non-irradiated organs, median follow-up 6.0 and 9.9 years | 6 years: 8% (events: 87/1110)  10 years: 11% (events: 121/1110) | 6 years: 11% (events: 122/1105)  10 years: 20% (events: 158/1105) | HR (95% CI): 0.74 (0.59, 0.94), P = 0.01 |
| *Harms & Toxicity* | | | | | |
| Acute toxicity, overall | NCT01266642  (Shaitelman, 2015) (22)  SOME CONCERNS | CTCAE v4.0, grade ≥ 2  < 3 months | 65/138 (47) | 116/149 (78) | P < 0.001 |
| Acute pneumonitis | NCT00793962  (Wang, 2019) (47)  LOW | CTCAE 3.0, grade 1-3 | Grade 1: 61/401 (15)  Grade 2:14/401 (3)  Grade 3: 0/401 | Grade 1: 62/409 (15)  Grade 2: 7/409 (2)  Grade 3: 0/409 | P = 0.28 |
|  | NCT01413269  (Wang, 2020) (48)  LOW | CTCAE 3.0, grade 2  < 3 months | 7/365 (2) | 11/363 (3) | P = 0.22 |
| Acute skin toxicity | DRKS00017763  (Schmeel, 2020) (35)  SOME CONCERNS | CTCAE v4.03, grade ≥ 2 | 19/70 (27) | 30/70 (43%) | OR (95% CI): 2.01 (0.99, 4.09)  P = 0.05 |
|  | NCT01266642  (Shaitelman, 2015) (22)  SOME CONCERNS | CTCAE v4.0, grade ≥ 2  < 3 months | 50/138 (36) | 103/149 (69) | P < 0.001 |
|  | NCT00156052  (Arsenault, 2020) (18)  SOME CONCERNS | ECOG, grade 2-3  At 4-6 weeks | 9/73 (12) | 28/73 (38) | P-value NR |
|  | NCT01413269  (Wang, 2020) (48)  LOW | CTCAE v3.0, grade 2-3  < 3 months | 11/365 (3) | 27/363 (0.7) | P = 0.02 |
|  | **TomoBreast**  NCT00459628  (Nan Parijs, 2012) (45)  SOME CONCERNS | RTOG, grade 2-3  Within 4 weeks | Grade 2: 10/37 (27)  Grade 3: 3/37 (8) | Grade 2: 7/32 (22)  Grade 3: 2/32 (6) | Comparison NR |
| Acute skin toxicity (undefined) | NCT00793962  (Wang, 2019) (47)  LOW | CTCAE 3.0, grade 1-3 | Grade 1-2: 351/401 (89)  Grade 3: 14/401 (3) | Grade 1-2: 357/401 (87)  Grade 3: 32/401 (8) | P < 0.0001 |
| Acute skin ulceration | NCT01266642  (Shaitelman, 2015) (22)  SOME CONCERNS | CTCAE v4.0, grade ≥ 2  < 3 months | 1/138 (1) | 2/149 (1) | P = 0.19 |
| Late toxicity, overall | NCT01266642  (Shaitelman, 2015) (22)  SOME CONCERNS | CTCAE v4.0, grade ≥ 2 6 months | 40/129 (31) | 46/142 (32) | P = 0.81 |
| Late dermatitis | NCT01266642  (Shaitelman, 2015) (22)  SOME CONCERNS | CTCAE v4.0, grade ≥ 2  6 months | 0/138 | 1/142 (1) | P = 0.73 |
| Late lymphedema | NCT00793962  (Wang, 2019) (47)  LOW | RTOG, grade 1-3 | Grade 1-2: 78/401 (19)  Grade 3: 3/401 (1) | Grade 1-2: 81/409 (20)  Grade 3: 3/409 (1) | P = 0.96 |
|  | NCT01266642  (Shaitelman, 2015) (22)  SOME CONCERNS | CTCAE v4.0, grade ≥ 2  6 months | 2/129 (2) | 7/142 (5) | P = 0.78 |
|  | NCT01413269  (Wang, 2020) (48)  LOW | RTOG, grade 2  >6 months | 2/365 (0.5) | 2/363 (0.6) | P = 0.74 |
| Late lung fibrosis | NCT01413269  (Wang, 2020) (48)  LOW | RTOG, grade 2  > 6 months | 0/365 | 1/363 (0.3) | P = 0.51 |
| Late pneumonitis | NCT01266642  (Shaitelman, 2015) (22)  SOME CONCERNS | CTCAE v4.0, grade ≥ 2  6 months | 0/129 | 0/142 | N/A |
| Late skin toxicity | NCT00156052  (Whelan, 2010) (19)  SOME CONCERNS | RTOG, grade 2 and 3  Over 5 years | 14/449 (3) | 14/424 (3) | P-value NR |
|  |  | RTOG, grade 2 and 3  Over 10 years | 21/235 (9) | 17/220 (8) | P-value NR |
|  | NCT00793962  (Wang, 2019) (47)  LOW | RTOG, grade 1-3  Median follow-up 58 months | Grade 1-2: 86/401 (21)  Grade 3: 1/401 (<1) | Grade 1-2: 90/409 (22)  Grade 3: 0/409 | P = 0.67 |
| Late skin ulceration | NCT01266642  (Shaitelman, 2015) (22)  SOME CONCERNS | CTCAE v4.0, grade ≥2  6 months | 0/129 | 0/142 | N/A |
| *Health-related Quality of Life (QoL)* | | | | | |
| Overall QoL | **BIG3-07/TROG 07.01**  NCT00470236  (King, 2020) (15)  SOME CONCERNS | EORTC QLQ-C30, overall score at 6 months, 1 year, and 2 years | Mean (SD):  6 months: 77.8 (18.2)  1 year: 79.2 (18.2)  2 years:78.9 (19.1) | Mean (SD):  6 months: 78.1 (18.2)  1 year: 78.0 (18.0)  2 years: 78.7 (18.9) | Comparison NR |
|  | NCT01266642  (Shaitelman, 2015 (22); Shaitelman, 2018 (23))  SOME CONCERNS | FACT-G and FACT-B v4, total mean scores at baseline, 6 months  FACT-B TOI v4, mean scores at baseline, 3 years | FACT-G:  Baseline: 92.8  6 months: 91.6  FACT-B:  Baseline: 120.1  6 months: 124.5  FACT-B TOI:  Baseline: 74.5  3 years: 77.9 | FACT-G:  Baseline: 91.6  6 months: 93.6  FACT-B:  Baseline: 118.8  6 months: 122.3  FACT-B TOI:  Baseline: 74.0  3 years: 77.6 | FACT-G:  Baseline: P = 0.35  6 months: P = 0.12  FACT-B:  Baseline: P = 0.46  6 months: P = 0.20  FACT-B TOI:  Baseline: P = 0.72  3 years: P = 0.20 |
| Global health status (QL) | **TomoBreast**  NCT00459628  (Versmessen, 2012) (44)  SOME CONCERNS | EORTC QLQ-C30, mean (SD) at baseline, end of radiation, 3 months, annually years 1-3 | Baseline: 67.2 (17.5)  End of therapy: 59.0 (2.9)  3 months: 65.8 (3.1)  1 year: 72.6 (3.1)  2 years: 76.2 (3.8)  3 years: 78.5 (5.3) | Baseline: 69.0 (21.7)  End of therapy: 67.0 (2.2)  3 months: 68.5 (2.2)  1 year: 72.3 (2.5)  2 years: 72.3 (3.2)  3 years: 74.4 (4.1) | Significant difference only at end of radiation (P = 0.029), otherwise NS (P-value NR) |
| Physical functioning |  |  | Baseline: 83.2 (16.0)  End of therapy: 79.4 (2.0)  3 months: 82.0 (2.2)  1 year: 83.6 (2.0)  2 years: 88.7 (1.9)  3 years: 89.9 (3.2) | Baseline: 84.1 (18.7)  End of therapy: 80.1 (1.6)  3 months: 80.7 (1.7)  1 year: 85.4 (2.0)  2 years: 84.1 (3.5)  3 years: 84.9 (3.3) | Differences NS (P-value NR) |
| Role functioning |  |  | Baseline: 66.4 (29.3)  End of therapy: 65.0 (4.2)  3 months: 75.8 (4.3)  1 year: 84.7 (4.5)  2 years: 94.1 (5.4)  3 years: 97.5 (8.7) | Baseline: 70.2 (27.4)  End of therapy: 66.9 (3.5)  3 months: 81.9 (4.6)  1 year: 79.9 (3.6)  2 years: 81.1 (4.3)  3 years: 80.3 (3.2) | Differences NS (P-value NR) |
| Emotional functioning |  |  | Baseline: 74.4 (20.0)  End of therapy: 75.4 (2.6)  3 months: 78.5 (2.7)  1 year: 77.3 (2.8)  2 years: 80.7 (4.1)  3 years: 81.3 (4.5) | Baseline: 78.8 (18.1)  End of therapy: 76.0 (2.5)  3 months: 75.6 (2.6)  1 year: 76.7 (3.5)  2 years: 76.7 (4.4)  3 years: 77.7 (6.2) | Differences NS (P-value NR) |
| Social functioning |  |  | Baseline: 82.2 (19.8)  End of therapy: 71.7 (3.1)  3 months: 82.6 (2.9)  1 year: 84.7 (3.7)  2 years: 90.5 (4.5)  3 years: 89.7 (7.0) | Baseline: 80.6 (22.6)  End of therapy: 78.6 (2.1)  3 months: 83.9 (2.6)  1 year: 89.4 (3.3)  2 years: 92.5 (6.2)  3 years:92.9 (7.4) | Differences NS (P-value NR) |

Supplemental Table 5. Detailed Results for Ultra-hypofractionation vs. Conventional or Moderate Hypofractionation Trials (Rated “Low” or “Some Concerns” Risk of Bias)

| **Outcome** | **Trial Name, Year**  **Trial #**  **Risk of Bias** | **Effect Measure/Definition** | **Intervention Arm**  **N Events/Total N (%)** | **Comparison Arm**  **N Events/Total N (%)** | **Results** |
| --- | --- | --- | --- | --- | --- |
| *Ultra-hypofractionation vs Conventional Radiation: Survival & Recurrence* | | | | | |
| Overall survival | **FAST**  NCT00107497  (FAST Trialists, 2011) (6)  LOW | Median follow-up at 3.1 years | Arm A (30 Gy): 98% (deaths: 5/305)  Arm B (28.5 Gy): 96% (deaths: 12/302) | 98% (deaths: 6/301) | Comparison NR |
| Breast-cancer-specific deaths | **FAST**  NCT00107497  (FAST Trialists, 2011) (6)  LOW | 10 years | Arm A (30 Gy): 1% (deaths: 2/305)  Arm B (28.5 Gy): 2% (deaths: 6/302) | 1% (deaths: 2/301) | Comparison NR |
| Local recurrence | **FAST**  NCT00107497  (Brunt, 2020 (7); FAST Trialists, 2011 (6))  LOW | Recurrence in ipsilateral breast and/or overlying skin, median follow-up at 3.1 and 9.9 years | Arm A (30 Gy):  3.1 years: 0% (events: 0/305)  9.9 years: 1% (events: 4/305)  Arm B (28.5 Gy):  3.1 years: 0% (events: 0/302)  9.9 years: 1% (events: 4/302) | 3.1 years: 1% (events: 2/301)  9.9 years: 1% (events: 3/301) | HR (95% CI):  Arm A (30 Gy):  1.36 (0.30, 6.06)  Arm B (28.5 Gy):  1.35 (0.30, 6.05) |
| Regional metastasis | **FAST**  NCT00107497  (FAST Trialists, 2011) (6)  LOW | Spread to axilla, supraclavicular fossa, and/or internal mammary chain, median follow-up at 3.1 years | Arm A (30 Gy): 0% (events: 0/305)  Arm B (28.5 Gy): 1% (events: 2/302) | 0.3% (events: 1/301) | Comparison NR |
| Distant metastasis | **FAST**  NCT00107497  (FAST Trialists, 2011) (6)  LOW |  | Arm A (30 Gy): 1% (events: 2/305)  Arm B (28.5 Gy): 3% (events: 10/302) | 2% (events: 5/301) | Comparison NR |
| *Ultra-hypofractionation vs Moderate Hypofractionation: Survival & Recurrence* | | | | | |
| Overall survival | **FAST-Forward**  ISRCTN19906132  (Brunt, 2020) (8)  LOW | Deaths any cause, 5 years (median follow-up 71.5 months) | Arm A (27 Gy): 92% (deaths: 105/1367)  Arm B (26 Gy): 93% (deaths: 90/1368) | 93% (deaths: 92/1361) | HR (95% CI):  Arm A (27 Gy): 1.12 (0.85, 1.48), P = 0.42  Arm B (26 Gy): 0.96 (0.72, 1.28), P = 0.78 |
| Local recurrence | **FAST-Forward**  ISRCTN19906132  (Brunt, 2020) (8)  LOW | Recurrence in ipsilateral breast, chest wall or skin, 5 years (median follow-up 71.5 months) | Arm A (27 Gy): 2% (events: 27/1367)  Arm B (26 Gy): 1% (events: 21/1368) | 2% (events: 31/1361) | HR (95% CI):  Arm A (27 Gy): 0.86 (0.51, 1.44), P = 0.56  Arm B (26 Gy): 0.67 (0.38, 1.16), P = 0.15 |
| Locoregional recurrence | **FAST-Forward**  ISRCTN19906132  (Brunt, 2020) (8)  LOW | Local or regional (axilla, supraclavicular fossa, and internal mammary chain), 5 years (median follow-up 71.5 months) | Arm A (27 Gy): 3% (events: 35/1367)  Arm B (26 Gy): 2% (events: 29/1368) | 3% (events: 43/1361) | HR (95% CI):  Arm A (27 Gy): 0.80 (0.51, 1.25), P = 0.33  Arm B (26 Gy): 0.66 (0.41, 1.06), P = 0.08 |
| Distant metastasis | **FAST-Forward**  ISRCTN19906132  (Brunt, 2020) (8)  LOW | 5 years (median follow-up 71.5 months) | Arm A (27 Gy): 5% (events: 69/1367)  Arm B (26 Gy): 6% (events: 76/1368) | 4% (events: 59/1361) | HR (95% CI):  Arm A (27 Gy): 1.16 (0.82, 1.64), P = 0.41  Arm B (26 Gy): 1.27 (0.90, 1.79), P = 0.17 |
| *Ultra-hypofractionation vs Conventional Radiation: Harms & Toxicity* | | | | | |
| Acute skin toxicity | **FAST**  NCT00107497  (FAST Trialists, 2011) (6)  LOW | RTOG, grade 2-4 | Arm A (30 Gy):  2: 13/111 (12)  3: 3/111 (3)  4: 0/111  Arm B (28.5 Gy):  2: 9/106 (9)  3: 2/106 (2)  4: 0/106 | 2: 39/110 (36)  3: 12/110 (11)  4: 0/110 | Comparison NR |
| *Ultra-hypofractionation vs Moderate Hypofractionation: Harms & Toxicity* | | | | | |
| Acute skin toxicity | **FAST-Forward**  ISRCTN19906132  (Brunt, 2016) (9)  LOW | RTOG, grade 2-3 (substudy 1)  Within 4 weeks | Arm A (27 Gy):  Grade 2: 20/51 (39)  Grade 3: 5/51 (10)  Arm B (26 Gy):  Grade 2: 14/52 (27)  Grade 3: 3/52 (6) | Grade 2: 24/44 (55)  Grade 3: 6/44 (14) | P-value NR |
|  |  | CTCAE v4.03, grade 2-3 (substudy 2)  Within 4 weeks | Arm A (27 Gy):  Grade 2: 11/41 (27)  Grade 3: 1/41 (2)  Arm B (26 Gy):  Grade 2: 19/53 (36)  Grade 3: 0/53 | Grade 2: 22/43 (51)  Grade 3: 0/43 | P-value NR |
|  | **YO-HAI5**  NCT03677427  (Van Hulle, 2021) (46)  SOME CONCERNS | CTCAE v4.03, grade 2  16.7 days ± 6.0 days post | 17/105 (16) | 11/94 (20) | P-value NR |
|  | **HYPORT**  NCT03788213  (Chakraborty, 2022 (12))  LOW | CTCAE v5.0, grade 2-3  23 weeks | Grade 2: 3/135 (2.2)  Grade 3: 1/135 (0.7) | Grade 2: 13/136 (9.6)  Grade 3: 2/136 (1.5) | P-value NR |
|  | **Trial Name/# NR**  (Ivanov, 2022 (13))  SOME CONCERNS | CTCAE v5.0 Grade 2  ≤ 6 months | 5/27 (18.5) | 1/33 (3) | P = 0.04 |
| Late skin toxicity | **Trial Name/# NR**  (Ivanov, 2022 (13))  SOME CONCERNS | RTOG/EORTC Grade ≥2  > 6 months | 0/27 | 0/33 | P-value NR |
| *Ultra-hypofractionation vs Moderate Hypofractionation: Health-related Quality of Life (QoL)* | | | | | |
| Overall QoL | **YO-HAI5**  NCT03677427  (Van Hulle, 2021) (46)  SOME CONCERNS | EORTC QLQ-C30/BR23, ≥ 10 pts decrease (from baseline)  16.7 days ±6.0 days post | Global score:  16/105 (15) | Global score:  30/94 (32) | P = 0.005 |
| Physical functioning |  |  | Physical functioning:  7/105 (7) | Physical functioning:  23/94 (24) | P = 0.0005 |
| Social functioning |  |  | Social functioning:  12/105 (11) | Social functioning  29/94 (31) | P =0.0007 |

Supplemental Table 6. Detailed Results for Accelerated Partial Breast Irradiation vs. Whole Breast Irradiation Trials (Rated “Low” or “Some Concerns” Risk of Bias)

| **Outcome** | **Trial Name, Year**  **Trial #**  **Risk of Bias** | **Effect Measure/Definition** | **Hypofractionation Arm**  **N Events/Total N (%)** | **Comparison Arm**  **N Events/Total N (%)** | **Results** |
| --- | --- | --- | --- | --- | --- |
| *Survival & Recurrence* | | | | | |
| Overall survival | **APBI-IMRT Florence**  NCT02104895  (Meattini, 2020) (2)  SOME CONCERNS | All cause deaths, 10 years | 5 years: 98% (deaths: 5/260)  7 years: 97% (deaths: 9/260)  10 years: 92% (deaths: 18/260) | 5 years: 97% (deaths: 8/260)  7 years: 94% (deaths: 15/260)  10 years: 92% (deaths: 20/260 | HR (95% CI): 0.95 (0.50, 1.79), P = 0.86 |
|  | **RAPID**  NCT00282035  (Whelan, 2019) (30)  SOME CONCERNS | Median follow-up 8.6 years | 93% (deaths: 76/1070) | 94% (deaths: 64/1065) | HR (95% CI): 1.18 (0.84, 1.64) |
| Breast-cancer-specific deaths | **APBI-IMRT Florence**  NCT02104895  (Meattini, 2020) (2)  SOME CONCERNS | 10 years | 5 years: 1% (deaths: 2/260)  7 years: 1% (deaths: 3/260)  10 years: 2% (deaths: 5/260) | 5 years: 1% (deaths: 3/260)  7 years: 2% (deaths: 6/260)  10 years: 3% (deaths: 8/260) | HR (95% CI): 0.65 (0.21, 1.99), P = 0.45 |
| Local recurrence | **APBI-IMRT Florence**  NCT02104895  (Meattini, 2020) (2)  SOME CONCERNS | Recurrence in ipsilateral breast, 10 years | 5 years: 2% (events: 6/260)  7 years: 3% (events: 7/260)  10 years: 4% (events: 9/260) | 5 years: 1% (events: 3/260)  7 years: 2% (events: 5/260)  10 years: 2% (events: 6/260) | HR (95% CI): 1.56 (0.55, 4.37), P = 0.40 |
|  | **RAPID**  NCT00282035  (Whelan, 2019) (30)  SOME CONCERNS | Recurrence in ipsilateral breast, median follow-up 8.6 years | 4% (events: 37/1070) | 3% (events: 28/1065) | HR (90% CI): 1.27 (0.84, 1.91) |
| Locoregional recurrence | **APBI-IMRT Florence**  NCT02104895  (Meattini, 2020) (2)  SOME CONCERNS | Includes recurrence in ipsilateral axillary, supraclavicular or internal mammary nodes, 10 years | 5 years: 2% (events: 6/260)  7 years: 3% (events: 7/260)  10 years: 4% (events: 9/260) | 5 years: 2% (events: 4/260)  7 years: 2% (events: 6/260)  10 years: 3% (events: 7/260) | HR (95% CI): 1.33 (0.49, 3.56), P = 0.58 |
| Distant metastasis | **APBI-IMRT Florence**  NCT02104895  (Meattini, 2020) (2)  SOME CONCERNS | Includes recurrence to distant organs (visceral and bone sites), 10 years | 5 years: 2% (events: 4/260)  7 years: 2% (events: 6/260)  10 years: 3% (events: 7/260) | 5 years: 3% (events: 8/260)  7 years: 6% (events: 15/260)  10 years: 8% (events: 20/260) | HR (95% CI): 0.89 (0.32, 2.47), P = 0.83 |
| *Toxicity & Harms* | | | | | |
| Acute toxicity, overall | **APBI-IMRT Florence**  NCT02104895  (Meattini, 2020) (2)  SOME CONCERNS | RTOG, grade ≥ 2  ≤ 6 months | 5/246 (2.0) | 98/260 (38) | P = 0.0001 |
|  | **RAPID**  NCT00282035  (Whelan, 2019) (30)  SOME CONCERNS | CTCAE v3, grade 2-3  ≤ 3 months | Grade 2: 281/1070 (26)  Grade 3: 19/1070 (2) | Grade 2: 466/1065 (44)  Grade 3: 18/1065 (2) | Grade ≥ 2: P < 0.0001 |
| Acute pneumonitis | **RAPID**  NCT00282035  (Whelan, 2019) (30)  SOME CONCERNS | CTCAE v3, grade 2-3  ≤ 3 months | Grade 2: 2/1070 (< 0.1)  Grade 3: 0/1070 | Grade 2: 7/1065 (0.7)  Grade 3: 1/1065 (< 0.1) | Comparison NR |
|  | NCT06007118  (Burkon, 2024 (25))  SOME CONCERNS | CTCAE, grade 2-3  ≤ 3 months | Grade 2: 0/42  Grade 3: 0/42 | Grade 2: 0/42  Grade 3: 0/42 | p-value N/A |
| Acute skin toxicity | **APBI-IMRT Florence**  NCT02104895  (Livi, 2015) (1)  SOME CONCERNS | RTOG, grade ≥ 2  ≤ 6 months | 5/246 (2) | 98/260 (38) | P = 0.0001 |
|  | **RAPID**  NCT00282035  (Whelan, 2019) (30)  SOME CONCERNS | CTCAE v3, grade 2-3  ≤ 3 months | Grade 2: 101/1070 (9)  Grade 3: 1/1070 (<0.1) | Grade 2: 322/1065 (30)  Grade 3: 6/1065 (0.6) | Comparison NR |
|  | NCT06007118  (Burkon, 2024 (25))  SOME CONCERNS | CTCAE, grade 2-3  ≤ 3 months | Grade 2: 0/42  Grade 3: 0/42 | Grade 2: 22/42 (52)  Grade 3: 1/42 (2.4) | Grade ≥ 2: P < 0.001 |
| Late toxicity, overall | **APBI-IMRT Florence**  NCT02104895  (Meattini, 2020) (2)  SOME CONCERNS | RTOG, grade ≥ 2  > 6 months to 10 years | 0/246 (0%) | 7/260 (3) | P = 0.02 |
|  | **RAPID**  NCT00282035  (Whelan, 2019 (30); Olivotto, 2013 (29))  SOME CONCERNS | CTCAE v3, grade 2-3  > 3 months through 3 and 8.6 years | 3 years:  Grade 2: 2/1070 (< 0.1)  Grade 3: 0/1070  8.6 years:  Grade 2: 298/1070 (28)  Grade 3: 48/1070 (5) | 3 years:  Grade 2: 2/1070 (< 0.1)  Grade 3: 0/1070  8.6 years:  Grade 2: 131/1065 (12)  Grade 3: 11/1065 (1) | Grade ≥ 2:  8.6 years: P < 0.0001 |
| Late pneumonitis | NCT06007118  (Burkon, 2024 (25))  SOME CONCERNS | CTCAE, grade 2-3  ≥ 6 months | Grade 2: 0/42  Grade 3: 0/42 | Grade 2: 0/42  Grade 3: 0/42 | p-value N/A |
| Late skin toxicity | **APBI-IMRT Florence**  NCT02104895  (Livi, 2015) (1)  SOME CONCERNS | RTOG, grade ≥ 2  > 6 months to 5 years | 0/246 | 2/260 (1) | P = 0.26 |
|  | NCT06007118  (Burkon, 2024 (25))  SOME CONCERNS | CTCAE, grade 2-3  ≥ 6 months | Grade 2: 0/42  Grade 3: 0/42 | Grade 2: 5/42 (12)  Grade 3: 0/42 | Grade ≥ 2: P = 0.026 |

Supplemental Table 7: PRISMA 2020 Checklist

| **Section and Topic** | **Item #** | **Checklist item** | **Location where item is reported** |
| --- | --- | --- | --- |
| **TITLE** | | |  |
| Title | 1 | Identify the report as a systematic review. | p. 1 |
| **ABSTRACT** | | |  |
| Abstract | 2 | See the PRISMA 2020 for Abstracts checklist. | p. 2 |
| **INTRODUCTION** | | |  |
| Rationale | 3 | Describe the rationale for the review in the context of existing knowledge. | p. 3 |
| Objectives | 4 | Provide an explicit statement of the objective(s) or question(s) the review addresses. | p. 3 |
| **METHODS** | | |  |
| Eligibility criteria | 5 | Specify the inclusion and exclusion criteria for the review and how studies were grouped for the syntheses. | p. 4: Study Selection; Supplemental Material 2 |
| Information sources | 6 | Specify all databases, registers, websites, organisations, reference lists and other sources searched or consulted to identify studies. Specify the date when each source was last searched or consulted. | p. 4: Data Sources and Searches |
| Search strategy | 7 | Present the full search strategies for all databases, registers and websites, including any filters and limits used. | Supplemental Table 1 |
| Selection process | 8 | Specify the methods used to decide whether a study met the inclusion criteria of the review, including how many reviewers screened each record and each report retrieved, whether they worked independently, and if applicable, details of automation tools used in the process. | p. 4: Study Selection |
| Data collection process | 9 | Specify the methods used to collect data from reports, including how many reviewers collected data from each report, whether they worked independently, any processes for obtaining or confirming data from study investigators, and if applicable, details of automation tools used in the process. | p. 4: Study Selection, Risk of Bias Assessment & Data Extraction |
| Data items | 10a | List and define all outcomes for which data were sought. Specify whether all results that were compatible with each outcome domain in each study were sought (e.g. for all measures, time points, analyses), and if not, the methods used to decide which results to collect. | p. 4: Risk of Bias Assessment & Data Extraction; Supplemental Table 2 |
|  | 10b | List and define all other variables for which data were sought (e.g. participant and intervention characteristics, funding sources). Describe any assumptions made about any missing or unclear information. | p. 4: Risk of Bias Assessment & Data Extraction; Supplemental Table 2 |
| Study risk of bias assessment | 11 | Specify the methods used to assess risk of bias in the included studies, including details of the tool(s) used, how many reviewers assessed each study and whether they worked independently, and if applicable, details of automation tools used in the process. | p. 4: Supplemental Table 3 |
| Effect measures | 12 | Specify for each outcome the effect measure(s) (e.g. risk ratio, mean difference) used in the synthesis or presentation of results. | p. 4: Synthesis |
| Synthesis methods | 13a | Describe the processes used to decide which studies were eligible for each synthesis (e.g. tabulating the study intervention characteristics and comparing against the planned groups for each synthesis (item #5)). | p. 4: Synthesis |
|  | 13b | Describe any methods required to prepare the data for presentation or synthesis, such as handling of missing summary statistics, or data conversions. | p. 4: Synthesis |
|  | 13c | Describe any methods used to tabulate or visually display results of individual studies and syntheses. | p. 4: Synthesis |
|  | 13d | Describe any methods used to synthesize results and provide a rationale for the choice(s). If meta-analysis was performed, describe the model(s), method(s) to identify the presence and extent of statistical heterogeneity, and software package(s) used. | p. 4: Synthesis |
|  | 13e | Describe any methods used to explore possible causes of heterogeneity among study results (e.g. subgroup analysis, meta-regression). | p. 4: Synthesis |
|  | 13f | Describe any sensitivity analyses conducted to assess robustness of the synthesized results. | N/A |
| Reporting bias assessment | 14 | Describe any methods used to assess risk of bias due to missing results in a synthesis (arising from reporting biases). | p. 4: Synthesis |
| Certainty assessment | 15 | Describe any methods used to assess certainty (or confidence) in the body of evidence for an outcome. | p. 4: Synthesis |
| **RESULTS** | | |  |
| Study selection | 16a | Describe the results of the search and selection process, from the number of records identified in the search to the number of studies included in the review, ideally using a flow diagram. | p. 5; Figure 1 |
|  | 16b | Cite studies that might appear to meet the inclusion criteria, but which were excluded, and explain why they were excluded. | Figure 1 |
| Study characteristics | 17 | Cite each included study and present its characteristics. | Table 2 |
| Risk of bias in studies | 18 | Present assessments of risk of bias for each included study. | Supplemental Table 3 |
| Results of individual studies | 19 | For all outcomes, present, for each study: (a) summary statistics for each group (where appropriate) and (b) an effect estimate and its precision (e.g. confidence/credible interval), ideally using structured tables or plots. | Table 3; Figures 1-3; Supplemental Table 4-6 |
| Results of syntheses | 20a | For each synthesis, briefly summarise the characteristics and risk of bias among contributing studies. | Results section |
|  | 20b | Present results of all statistical syntheses conducted. If meta-analysis was done, present for each the summary estimate and its precision (e.g. confidence/credible interval) and measures of statistical heterogeneity. If comparing groups, describe the direction of the effect. | Table 3; Figures 1-3 |
|  | 20c | Present results of all investigations of possible causes of heterogeneity among study results. | Figures 1-3 |
|  | 20d | Present results of all sensitivity analyses conducted to assess the robustness of the synthesized results. | N/A |
| Reporting biases | 21 | Present assessments of risk of bias due to missing results (arising from reporting biases) for each synthesis assessed. | Table 3 |
| Certainty of evidence | 22 | Present assessments of certainty (or confidence) in the body of evidence for each outcome assessed. | Table 3 |
| **DISCUSSION** | | |  |
| Discussion | 23a | Provide a general interpretation of the results in the context of other evidence. | p. 9 |
|  | 23b | Discuss any limitations of the evidence included in the review. | p. 9-10 |
|  | 23c | Discuss any limitations of the review processes used. | p. 10 |
|  | 23d | Discuss implications of the results for practice, policy, and future research. | p. 10-11 |
| **OTHER INFORMATION** | | |  |
| Registration and protocol | 24a | Provide registration information for the review, including register name and registration number, or state that the review was not registered. | p. 3 |
|  | 24b | Indicate where the review protocol can be accessed, or state that a protocol was not prepared. | p. 3 |
|  | 24c | Describe and explain any amendments to information provided at registration or in the protocol. | N/A |
| Support | 25 | Describe sources of financial or non-financial support for the review, and the role of the funders or sponsors in the review. | p. 19 |
| Competing interests | 26 | Declare any competing interests of review authors. | p. 19 |
| Availability of data, code and other materials | 27 | Report which of the following are publicly available and where they can be found: template data collection forms; data extracted from included studies; data used for all analyses; analytic code; any other materials used in the review. | p. 19 |

*From:* Page MJ, McKenzie JE, Bossuyt PM, Boutron I, Hoffmann TC, Mulrow CD, et al. The PRISMA 2020 statement: an updated guideline for reporting systematic reviews. BMJ 2021;372:n71. doi: 10.1136/bmj.n71. This work is licensed under CC BY 4.0. To view a copy of this license, visit <https://creativecommons.org/licenses/by/4.0/>

References

1.	Livi L, Meattini I, Simontacchi G, Paiar F, Scotti V, De Luca Cardillo C, et al. Accelerated partial breast irradiation using intensity-modulated radiotherapy versus whole breast irradiation: 5-year survival analysis of a phase 3 randomised controlled trial. European Journal of Cancer. 2015;51(4):451-63.

2.	Meattini I, Lucidi S, Marrazzo L, Desideri I, Scotti V, Simontacchi G, et al. Accelerated partial-breast irradiation compared with whole-breast irradiation for early breast cancer: Long-term results of the randomized phase III APBI-IMRT-florence trial. Journal of Clinical Oncology. 2020;38(35):4175-83.

3.	Meattini I, Di Brina L, Mangoni M, Meacci F, Francolini G, Desideri I, et al. Accelerated partial breast irradiation using intensity-modulated radiotherapy technique compared to whole breast irradiation for patients aged 70 years or older: subgroup analysis from a randomized phase 3 trial. Breast Cancer Research and Treatment. 2015;153(3):539-47.

4.	Baillet F, Housset M, Maylin C, Boisserie G, Bettahar R, Delanian S, et al. The use of a specific hypofractionated radiation therapy regimen versus classical fractionation in the treatment of breast cancer: a randomized study of 230 patients. International journal of radiation oncology, biology, physics. 1990;19(5):1131-3.

5.	Das P, Das T, Jana A, Gupta P, Gupta P, Das S. Comparison of result and outcome of conventional and hypofractionated radiotherapy in post-operative breast cancer patients. International Journal of Medical Science and Public Health. 2018;7(6):452-6.

6.	FAST Trialists Group, Agrawal RK, Alhasso A, Barrett-Lee PJ, Bliss JM, Bliss P, et al. First results of the randomised UK FAST Trial of radiotherapy hypofractionation for treatment of early breast cancer (CRUKE/04/015). Radiotherapy and oncology: Journal of the European Society for Therapeutic Radiology and Oncology. 2011;100(1):93-100.

7.	Brunt AM, Haviland JS, Sydenham M, Agrawal RK, Algurafi H, Alhasso A, et al. Ten-Year Results of FAST: A Randomized Controlled Trial of 5-Fraction Whole-Breast Radiotherapy for Early Breast Cancer. Journal of clinical oncology : official journal of the American Society of Clinical Oncology. 2020;38(28):3261-72.

8.	Murray Brunt A, Haviland JS, Wheatley DA, Sydenham MA, Alhasso A, Bloomfield DJ, et al. Hypofractionated breast radiotherapy for 1 week versus 3 weeks (FAST-Forward): 5-year efficacy and late normal tissue effects results from a multicentre, non-inferiority, randomised, phase 3 trial. Lancet (London, England). 2020;395(10237):1613-26.

9.	Brunt AM, Wheatley D, Yarnold J, Somaiah N, Kelly S, Harnett A, et al. Acute skin toxicity associated with a 1-week schedule of whole breast radiotherapy compared with a standard 3-week regimen delivered in the UK FAST-Forward Trial. Radiotherapy and oncology : journal of the European Society for Therapeutic Radiology and Oncology. 2016;120(1):114-8.

10.	Hosseini S, Shahabadi M, Salek R, Fanipakdel A, Ghofraniha L, Ghaderi F, et al. Accelerated hypofractionated whole breast radiotherapy for early breast cancer; arandomized phase iii clinical trial. Acta Medica Iranica. 2019;57(11):645-52.

11.	Hou H-L, Song Y-C, Li R-Y, Zhu L, Zhao L-J, Yuan Z-Y, et al. Similar Outcomes of Standard Radiotherapy and Hypofractionated Radiotherapy Following Breast-Conserving Surgery. Medical science monitor : international medical journal of experimental and clinical research. 2015;21:2251-6.

12.	Chakraborty S, Chatterjee S. HYPORT adjuvant acute toxicity and patient dosimetry quality assurance results - Interim analysis. Radiotherapy and oncology : journal of the European Society for Therapeutic Radiology and Oncology. 2022;174:59-68.

13.	Ivanov O, Milovancev A, Petrovic B, Prvulovic Bunovic N, Licina J, Bojovic M, et al. Ultra-Hypofractionated vs. Moderate Fractionated Whole Breast Three Dimensional Conformal Radiotherapy during the COVID-19 Pandemic. Medicina (Kaunas, Lithuania). 2022;58(6).

14.	Kalita AK, Bhattacharyya M, Jagtap VK, Sunku R, Medhi PP, Hasan FA, et al. Radiotherapy in Post Mastectomy High Risk Breast Cancer: Early results of a Prospective Study comparing Conventional versus Hypofractionated Radiotherapy. Journal of Medical Science and Clinical Research. 2018;6(7):743-51.

15.	King MT, Link EK, Whelan TJ, Olivotto IA, Kunkler I, Westenberg AH, et al. Quality of life after breast-conserving therapy and adjuvant radiotherapy for non-low-risk ductal carcinoma in situ (BIG 3-07/TROG 07.01): 2-year results of a randomised, controlled, phase 3 trial. The Lancet Oncology. 2020;21(5):685-98.

16.	Kumbhaj P, Sharma R, Saini P, Patel P. Study of two different dose fractionation schedules of post mastectomy chest wall irradiation in carcinoma breast patients. International Journal of Medical Science and Public Health. 2013;2(4):1001-5.

17.	Maiti S, Meyur S, Mandal BC, Shenoi LR, Biswas S, Basu S. Comparison of conventional and hypofractionated radiation after mastectomy in locally advanced breast cancer: A prospective randomised study on dosimetric evaluation and treatment outcome. Journal of Radiotherapy in Practice. 2021;20(1):30-8.

18.	Arsenault J, Parpia S, Goldberg M, Rakovitch E, Reiter H, Doherty M, et al. Acute Toxicity and Quality of Life of Hypofractionated Radiation Therapy for Breast Cancer. International journal of radiation oncology, biology, physics. 2020;107(5):943-8.

19.	Whelan TJ, Pignol J-P, Levine MN, Julian JA, MacKenzie R, Parpia S, et al. Long-term results of hypofractionated radiation therapy for breast cancer. The New England journal of medicine. 2010;362(6):513-20.

20.	Whelan T, MacKenzie R, Julian J, Levine M, Shelley W, Grimard L, et al. Randomized trial of breast irradiation schedules after lumpectomy for women with lymph node-negative breast cancer. Journal of the National Cancer Institute. 2002;94(15):1143-50.

21.	Weng JK, Lei X, Schlembach P, Bloom ES, Shaitelman SF, Arzu IY, et al. Five-Year Longitudinal Analysis of Patient-Reported Outcomes and Cosmesis in a Randomized Trial of Conventionally Fractionated Versus Hypofractionated Whole-Breast Irradiation. International journal of radiation oncology, biology, physics. 2021;111(2):360-70.

22.	Shaitelman SF, Schlembach PJ, Arzu I, Ballo M, Bloom ES, Buchholz D, et al. Acute and Short-term Toxic Effects of Conventionally Fractionated vs Hypofractionated Whole-Breast Irradiation: A Randomized Clinical Trial. JAMA oncology. 2015;1(7):931-41.

23.	Shaitelman SF, Lei X, Thompson A, Schlembach P, Bloom ES, Arzu IY, et al. Three-year outcomes with hypofractionated versus conventionally fractionated whole-breast irradiation: Results of a randomized, noninferiority clinical trial. Journal of Clinical Oncology. 2018;36(35):3495-503.

24.	Laughlin BS, Corbin KS, Toesca DAS, Thorpe CS, Golafshar MA, Pockaj B, et al. Physician- and Patient-Reported Outcomes of the MC1635 Phase 3 Trial of Ultrahypofractionated Versus Moderately Hypofractionated Adjuvant Radiation Therapy After Breast-Conserving Surgery. International journal of radiation oncology, biology, physics. 2024;118(4):1049-59.

25.	Burkon P, Selingerova I, Slavik M, Holanek M, Vrzal M, Coufal O, et al. Toxicity of external beam accelerated partial-breast irradiation (APBI) in adjuvant therapy of early-stage breast cancer: prospective randomized study. Radiation oncology (London, England). 2024;19(1):17.

26.	Gil GOB, de Andrade WP, Diniz PHC, Cantidio FS, Queiroz IN, Gil MLBV, et al. A phase II randomized clinical trial to assess toxicity and quality of life of breast cancer patients with hypofractionated versus conventional fractionation radiotherapy with regional nodal irradiation in the context of COVID-19 crisis. Frontiers in Oncology. 2023;13:1202544.

27.	Offersen BV, Alsner J, Nielsen HM, Jakobsen EH, Nielsen MH, Krause M, et al. Hypofractionated Versus Standard Fractionated Radiotherapy in Patients With Early Breast Cancer or Ductal Carcinoma In Situ in a Randomized Phase III Trial: The DBCG HYPO Trial. Journal of clinical oncology : official journal of the American Society of Clinical Oncology. 2020;38(31):3615-25.

28.	Owen JR, Ashton A, Bliss JM, Homewood J, Harper C, Hanson J, et al. Effect of radiotherapy fraction size on tumour control in patients with early-stage breast cancer after local tumour excision: long-term results of a randomised trial. The Lancet Oncology. 2006;7(6):467-71.

29.	Olivotto IA, Berrang T, Truong PT, Beckham W, Nichol A, Reed M, et al. Interim cosmetic and toxicity results from RAPID: A randomized trial of accelerated partial breast irradiation using three-dimensional conformal external beam radiation therapy. Journal of Clinical Oncology. 2013;31(32):4038-45.

30.	Whelan TJ, Julian JA, Berrang TS, Kim DH, Germain I, Nichol AM, et al. External beam accelerated partial breast irradiation versus whole breast irradiation after breast conserving surgery in women with ductal carcinoma in situ and node-negative breast cancer (RAPID): a randomised controlled trial. Lancet. 2019;394(10215):2165-72.

31.	Purohit R, Sharma N, Kumar R, Jakhar SL. Comparison of Acute Toxicities in Conventional and Hypofractionated Radiotherapy in Post-Mastectomy Breast Cancer. Journal of Medical Science and Clinical Research. 2016;4(6):10721-4.

32.	Rastogi K, Jain S, Bhatnagar AR, Bhaskar S, Gupta S, Sharma N. A Comparative Study of Hypofractionated and Conventional Radiotherapy in Postmastectomy Breast Cancer Patients. Asia-Pacific Journal of Oncology Nursing. 2017;5(1):107-13.

33.	Li X, Sanz J, Foro P, Martinez A, Zhao M, Reig A, et al. Long-term results of a randomized partial irradiation trial compared to whole breast irradiation in the early stage and low-risk breast cancer patients after conservative surgery. Clinical & translational oncology : official publication of the Federation of Spanish Oncology Societies and of the National Cancer Institute of Mexico. 2021;23(10):2127-32.

34.	Rodriguez N, Sanz X, Dengra J, Foro P, Membrive I, Reig A, et al. Five-year outcomes, cosmesis, and toxicity with 3-dimensional conformal external beam radiation therapy to deliver accelerated partial breast irradiation. International journal of radiation oncology, biology, physics. 2013;87(5):1051-7.

35.	Schmeel LC, Koch D, Schmeel FC, Rohner F, Schoroth F, Bucheler BM, et al. Acute radiation-induced skin toxicity in hypofractionated vs. conventional whole-breast irradiation: An objective, randomized multicenter assessment using spectrophotometry. Radiotherapy and oncology : journal of the European Society for Therapeutic Radiology and Oncology. 2020;146:172-9.

36.	Shahid A, Athar MA, Asghar S, Zubairi T, Murad S, Yunas N. Post mastectomy adjuvant radiotherapy in breast cancer: a comparision of three hypofractionated protocols. JPMA The Journal of the Pakistan Medical Association. 2009;59(5):282-7.

37.	Le Bras A, Belkacemi Y, Bourgier C, Gabelle-Flandin I, Petit A, Guilbert P, et al. Economic evaluation and budget-impact of accelerated partial breast irradiation (APBI) versus standard or hypofractionated whole breast irradiation (WBI) in postmenopausal women with early-stage breast cancer. Results from the French SHARE randomized trial. Radiotherapy and oncology: Journal of the European Society for Therapeutic Radiology and Oncology. 2023;187:109818.

38.	Spooner D, Stocken DD, Jordan S, Bathers S, Dunn JA, Jevons C, et al. A randomised controlled trial to evaluate both the role and the optimal fractionation of radiotherapy in the conservative management of early breast cancer. Clinical oncology (Royal College of Radiologists (Great Britain)). 2012;24(10):697-706.

39.	START Trialists Group, Bentzen SM, Agrawal RK, Aird EGA, Barrett JM, Barrett-Lee PJ, et al. The UK Standardisation of Breast Radiotherapy (START) Trial A of radiotherapy hypofractionation for treatment of early breast cancer: a randomised trial. The Lancet Oncology. 2008;9(4):331-41.

40.	START Trialists Group, Bentzen SM, Agrawal RK, Aird EGA, Barrett JM, Barrett-Lee PJ, et al. The UK Standardisation of Breast Radiotherapy (START) Trial B of radiotherapy hypofractionation for treatment of early breast cancer: a randomised trial. Lancet (London, England). 2008;371(9618):1098-107.

41.	Haviland JS, Owen JR, Dewar JA, Agrawal RK, Barrett J, Barrett-Lee PJ, et al. The UK Standardisation of Breast Radiotherapy (START) trials of radiotherapy hypofractionation for treatment of early breast cancer: 10-year follow-up results of two randomised controlled trials. The Lancet Oncology. 2013;14(11):1086-94.

42.	Swanick CW, Lei X, Shaitelman SF, Schlembach PJ, Bloom ES, Fingeret MC, et al. Longitudinal analysis of patient-reported outcomes and cosmesis in a randomized trial of conventionally fractionated versus hypofractionated whole-breast irradiation. Cancer. 2016;122(18):2886-94.

43.	Taher AN, El-Baradie MM, Essa H, Zaki OE, Ezzat S. Hypofractionation versus Conventional Fractionation Radiotherapy after Conservative Treatment of Breast Cancer: Early Skin Reactions and Cosmetic Results. Journal of the Egyptian National Cancer Institute. 2004;16(3):178-87.

44.	Versmessen H, Vinh-Hung V, Van Parijs H, Miedema G, Voordeckers M, Adriaenssens N, et al. Health-related quality of life in survivors of stage I-II breast cancer: randomized trial of post-operative conventional radiotherapy and hypofractionated tomotherapy. BMC Cancer. 2012;12:495.

45.	Van Parijs H, Miedema G, Vinh-Hung V, Verbanck S, Adriaenssens N, Kerkhove D, et al. Short course radiotherapy with simultaneous integrated boost for stage I-II breast cancer, early toxicities of a randomized clinical trial. Radiation oncology (London, England). 2012;7:80.

46.	Van Hulle H, Vakaet V, Monten C, Deseyne P, Schoepen M, Colman C, et al. Acute toxicity and health-related quality of life after accelerated whole breast irradiation in 5 fractions with simultaneous integrated boost. Breast (Edinburgh, Scotland). 2021;55:105-11.

47.	Wang S-L, Fang H, Song Y-W, Wang W-H, Hu C, Liu Y-P, et al. Hypofractionated versus conventional fractionated postmastectomy radiotherapy for patients with high-risk breast cancer: a randomised, non-inferiority, open-label, phase 3 trial. The Lancet Oncology. 2019;20(3):352-60.

48.	Wang S-L, Fang H, Hu C, Song Y-W, Wang W-H, Jin J, et al. Hypofractionated Versus Conventional Fractionated Radiotherapy After Breast-Conserving Surgery in the Modern Treatment Era: A Multicenter, Randomized Controlled Trial From China. Journal of clinical oncology : official journal of the American Society of Clinical Oncology. 2020;38(31):3604-14.

49.	Yadav BS, Loganathan S, Sharma SC, Singh R, Dahiya D. Comparison of Toxicity and Cosmetic Outcomes After Accelerated Partial Breast Irradiation or Whole Breast Irradiation Using 3-Dimensional Conformal External Beam Radiation Therapy. Advances in Radiation Oncology. 2020;5(2):171-9.

50.	Zhao XB, Ren GS. Analysis of radiotherapy optimization regimens after modified radical mastectomy. European review for medical and pharmacological sciences. 2016;20(22):4705-9.

51.	Zhao S, Huang F, Chen X, Cao X, Yu J, Liu Y. The long-term outcome of adjuvant hypofractionated radiotherapy and conventional fractionated radiotherapy after breastconserving surgery for early breast cancer: A prospective analysis of 107 cases. Journal of Thoracic Disease. 2017;9(10):3840-50.

52.	Van Parijs H, Cecilia-Joseph E, Gorobets O, Storme G, Adriaenssens N, Heyndrickx B, et al. Lung-heart toxicity in a randomized clinical trial of hypofractionated image guided radiation therapy for breast cancer. Frontiers in Oncology. 2023;13:1211544.

53.	Goldberg M, Parpia S, Rakovitch E, Chang L, Bowen J, Lukka H, et al. Long-term outcomes and effects of hypofractionated radiotherapy in microinvasive breast cancer: Analysis from a randomized trial. Breast (Edinburgh, Scotland). 2023;68:189-93.
